# Effect of Norovirus Inoculum Dose on Virus Kinetics, Shedding, and Symptoms

**DOI:** 10.3201/eid2907.230117

**Published:** 2023-07

**Authors:** Yang Ge, W. Zane Billings, Antone Opekun, Mary Estes, David Graham, Juan Leon, Katia Koelle, Ye Shen, Robert Atmar, Benjamin Lopman, Andreas Handel

**Affiliations:** University of Southern Mississippi School of Health Professions, Hattiesburg, Mississippi, USA (Y. Ge);; University of Georgia, Athens, Georgia, USA (W.Z. Billings, Y. Shen, A. Handel);; Baylor College of Medicine, Houston, Texas, USA (A. Opekun, M. Estes, D. Graham, R. Atmar);; Emory University, Atlanta, Georgia, USA (J. Leon, K. Koelle, B. Lopman);

**Keywords:** norovirus, inoculum dose, virus shedding, kinetics, viruses, foodborne disease, food safety

## Abstract

The effect of norovirus dose on outcomes such as virus shedding and symptoms after initial infection is not well understood. We performed a secondary analysis of a human challenge study by using Bayesian mixed-effects models. As the dose increased from 4.8 to 4,800 reverse transcription PCR units, the total amount of shed virus in feces increased from 4.5 × 10^11^ to 3.4 × 10^12^ genomic equivalent copies; in vomit, virus increased from 6.4 × 10^5^ to 3.0 × 10^7^ genomic equivalent copies. Onset time of viral shedding in feces decreased from 1.4 to 0.8 days, and time of peak viral shedding decreased from 2.3 to 1.5 days. Time to symptom onset decreased from 1.5 to 0.8 days. One type of symptom score increased. An increase in norovirus dose was associated with more rapid shedding and symptom onset and possibly increased severity. However, the effect on virus load and shedding was inconclusive.

Norovirus is a major cause of foodborne disease and causes a large number of cases, hospitalizations, and deaths in the United States and globally (*1*–*4*). Specific treatments are not available, and vaccines are still under development (*4*,*5*). Generic infection control measures are the best approaches to minimizing disease burden (*6*–*10*).

An increase in exposure dose (number of virus particles) is associated with an increased risk for infection; this principle applies to norovirus (*11*–*14*) and many other pathogens (*15*,*16*). Less is known about the possible effect of dose on infection outcomes after infection has occurred. For acute infections such as influenza, infectious bronchitis virus, and parainfluenza virus, animal studies and models suggest that dose influences the virus load kinetics (*17*–*19*). For norovirus, some evidence from experimental challenge studies suggests that dose is associated with more rapid onset of symptoms (*20*). To further elucidate the effect of inoculum dose on infection outcomes such as virus shedding and symptom severity, we performed a secondary analysis of data from a human norovirus challenge study (*20*).

## Methods

In this article, we will give brief descriptions of our methods. We have also provided complete modeling and analysis details, including all data and code needed to reproduce our results (Appendix).

### Data

The data we used for our analyses are from a human challenge study registered at ClinicalTrials.gov (trial no. NCT00138476) (*20*–*24*). The clinical protocol was reviewed and approved by the institutional review boards of the Baylor College of Medicine and The Houston Methodist Hospital, and written informed consent was obtained from each study participant.

In the challenge study, 57 healthy persons (18–50 years of age) were randomly inoculated with either placebo or norovirus genogroup I genotype 1 strain (GI.1 NV) at 4 different doses (0.48, 4.8, 48, or 4,800 reverse transcription PCR [RT-PCR] units). Of the 21 persons who became infected, 1 person was unavailable for follow-up, and thus we excluded that patient from all analyses. In addition, only 1 person in the 0.48-unit dose group became infected, so we excluded this person from our main analyses. Therefore, remaining for our analysis were 6 persons in the 4.8-unit dose group, 7 persons in the 4.8-unit dose group, and 6 persons in the 4,800-unit dose group. We provide analyses that include the 1 person who was infected at the 0.48-dose level (Appendix).

All persons were isolated in the research center for >4 days (96 hours) after inoculation. The study personnel collected samples of feces and vomit and recorded clinical symptoms.; samples were also collected for 4–8 weeks postinoculation. For some of our analyses, we focused on the 96 hours during which persons were under clinical observation. For other analyses, we included the data collected after persons returned home. We state which data are used for each analysis.

### Overall Analysis Approach and Implementation

Because we performed a secondary data analysis, a strict null hypothesis significance testing framework using p values was not suitable, so we performed all analyses in a Bayesian framework. For all analyses, we used Bayesian mixed-effects models. We treated the dose as a continuous variable for the results shown in the article. We also provide a sensitivity analysis with dose modeled as categorical (Appendix). We report the mean of the estimated posterior distribution with 95% equal-tailed credible intervals (CrIs) for all model results (*25*). We conducted all analyses using R version 4.2.3 (*26*), and Stan (*27*), accessed through the brms package in R (*28*). We used Rhat values to diagnose convergence (*28*). 

### Analysis of Virus Shedding Outcomes

We measured virus shedding concentration in samples by either an immunomagnetic capture (IMC) RT-PCR, which provided a qualitative readout (positive or negative), or real-time quantitative RT-PCR (qRT-PCR), which provided a quantitative readout in genomic equivalent copies (GEC) (*21*). Those 2 methods had limits of detection (LOD) at 15,000 GEC/g of stool (LOD1) and 40,000,000 GEC/g of stool (LOD2). Therefore, the virus shedding concentration could be between 0 and LOD1 (negative IMC, negative qRT-PCR), between LOD1 and LOD2 (positive IMC, negative qRT-PCR), or a quantitative measurement above LOD2 (positive qRT-PCR). We reported vomit shedding data similarly, with either a numeric value or a positive or negative readout. We accounted for this censored data structure in our models (Appendix).

We obtained the total virus contained in each sample by multiplying virus concentration by sample weight for feces (GEC/g × weight of feces in grams) or sample volume for vomit (GEC/mL × volume of vomit in mL). We calculated each participant’s total amount of virus shedding in feces and vomit by summing virus shedding values for all samples per participant. We used a linear model structure to analyze associations between inoculum dose and the total amount of virus shedding.

**Figure 5:**
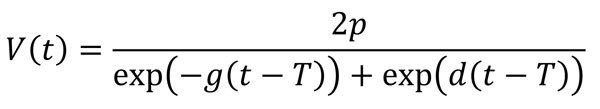
4-parameter equation modeling the longitudinal time-series of virus concentration in feces, V(t).

In a further analysis, we modeled the longitudinal time-series of virus concentration in feces, *V*(*t*), using the 4-parameter equation (Figure 5)

which was shown to accurately describe trajectories of acute viral infections (*17*,*29*). We fitted the trajectories by using a Bayesian nonlinear mixed-effects model in which the mean of the response was described using this equation. We used the comparison between the parameter’s prior and posterior distributions to ensure that the choice of prior distribution had no significant effect on our results. We sampled from the posterior distribution of the estimated parameters to obtain predicted trajectories of virus concentration kinetics. From those time-series, we computed several summary quantities: virus shedding onset (time at which the trajectory crossed LOD1); time to peak virus shedding; shedding duration, defined as the total amount of time at which virus concentration was above LOD1; and total amount of virus shed, defined as the area under the virus concentration curve.

Vomiting episodes were too few (11 persons with 26 samples of vomit) to enable a time-series analysis similar to the one we performed for virus shedding in feces. We have compiled vomit event time-series data (Appendix).

### Analysis of Symptom Outcomes

The study recorded 10 kinds of symptoms: body temperature, malaise, muscle aches, headache, nausea, chills, anorexia, cramps, unformed or liquid feces, and vomiting. Clinical symptom scores (except feces and vomit) were reported as none = 0, mild = 1, moderate = 2, or severe = 3. For feces, we used a scoring of solid = 0, unformed = 1, and liquid = 2. Vomit was reported as absent = 0 or present = 1.

We considered time to symptom onset (incubation period) and 2 symptom scores as outcomes of interest. We defined time to system onset as the time from inoculation to the first reported symptom of any type. For the first symptom score, we used a modified Vesikari score (MVS) that was previously applied to measure norovirus severity (*5*,*30*–*33*). We computed the MVS by using a limited number of symptoms (i.e., fever, diarrhea, and vomiting). We also developed an additional score, which we call the comprehensive symptom score (CSS), which encompasses all reported 10 symptoms in this study. Additional details of score computation, scores for each individual, and further model details are provided (Appendix).

### Sensitivity Analyses

We performed 2 sensitivity analyses. In the first analysis, we treated dose as categorical rather than continuous. In the second analysis, we included 1 person who became infected after exposure to a dose of 0.48 RT-PCR units. 

## Results

### Data Description

Detailed descriptions of the study can be found in previous publications (*20*–*24*). We summarized characteristics of the infected persons included in our analyses (Table). Distributions of age, sex, and ABO blood group status were generally similar across dose groups.

**Table:** Selected characteristics of patients in in study of the effect of norovirus inoculum dose on virus kinetics, shedding, and symptoms*

Characteristic	Dose, RT-PCR units			
	0.48	4.8	48	4,800
No. participants	1	6	7	6
Age, y, median (range)	24 (24–24)	30 (21–39)	24 (22–34)	28 (22–47)
Sex				
F	1 (100)	2 (33)	4 (57)	2 (33)
M	0	4 (67)	3 (43)	4 (67)
Blood type group				
A	0	2 (33)	2 (29)	3 (50)
O	1 (100)	4 (67)	5 (71)	3 (50)

*Values are no. (%) except as indicated. RT-PCR, reverse transcription PCR.

### Association between Dose and Total Virus Shedding

**Figure 1:**
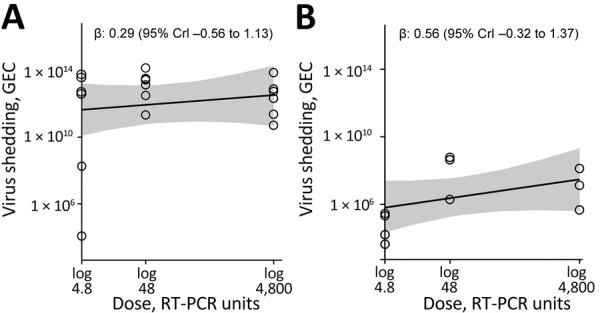
Total virus shedding in feces and vomit of patients challenged with norovirus in study of the effect of norovirus inoculum dose on virus kinetics, shedding, and symptoms. A) Cumulative virus shedding in feces. B) Cumulative virus shedding in vomit. Open circles represent raw data points. Lines and shaded regions indicate means and 95% CrIs of the fitted Bayesian model. Missing values attributable to limits of detection were replaced with fixed values (Appendix). CrI, credible interval; GEC, genomic equivalent copies; RT-PCR, reverse transcription PCR.

We computed total virus shedding in either feces or vomit by summing the amount of shed virus in all samples for each person. We focused on fecal shedding during the first 96 hours of the study, when patients were under clinical observation. Almost all viral shedding events that occurred during this timeframe were recorded. Every person shed virus in >1 fecal sample. All vomiting events occurred within the first 96 hours, and only 11 persons vomited. Virus shedding showed some association with dose, although with a fair amount of uncertainty (Figure 1), leading to overall inconclusive results. We developed an alternative analysis using fecal shedding that includes the self-reported data after persons returned to their homes (Appendix). In that case, we observed no noticeable association.

### Association between Dose and Viral Kinetics

Next, we fitted the virus concentration model to the time-series data for virus load for each person. The parameter’s prior and posterior distributions showed that the choice of prior distribution had no significant effect on our results (Appendix).

**Figure 2:**
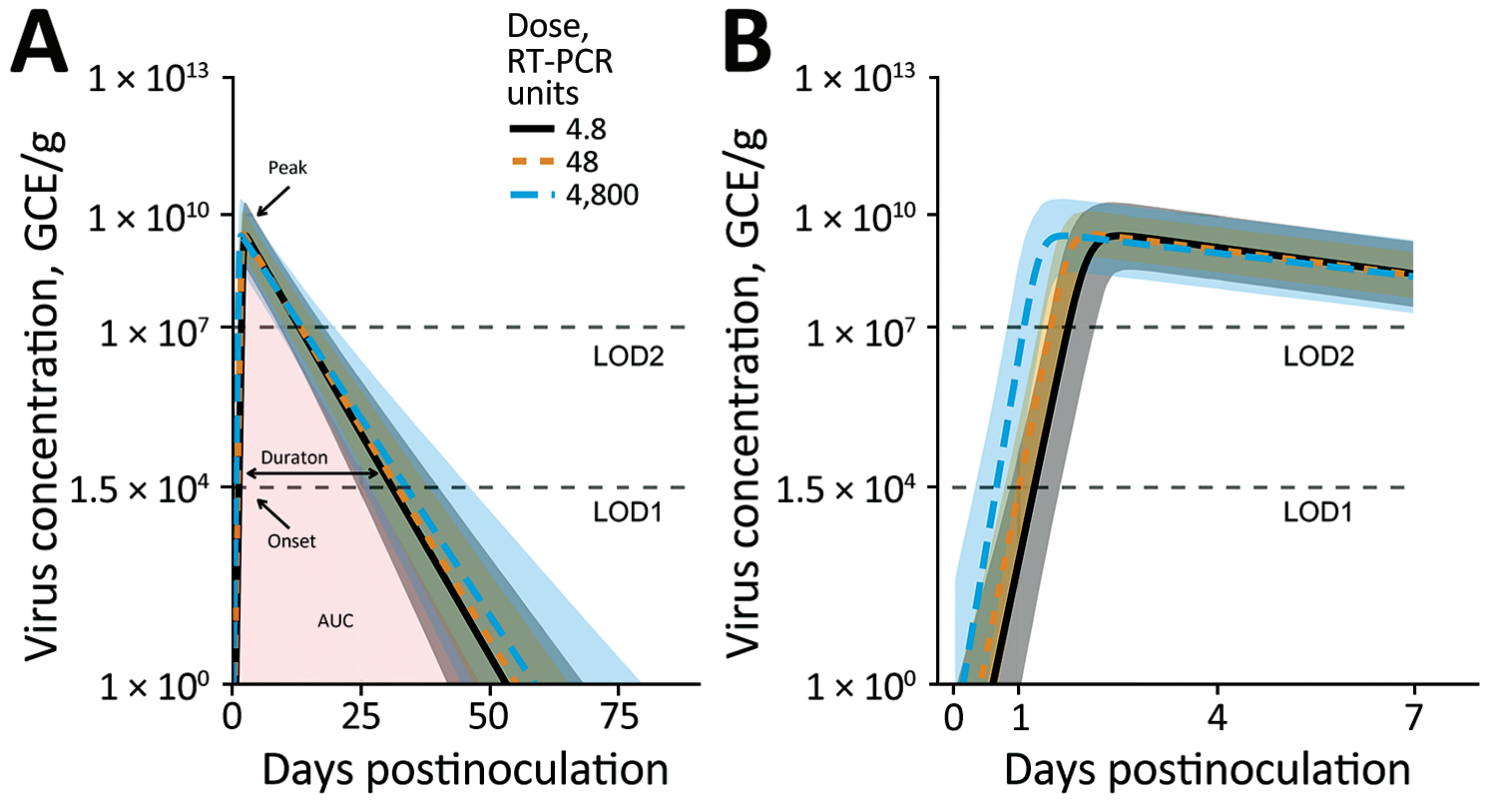
Fitted virus concentration (GEC/g) in feces of patients challenged with norovirus in study of the effect of norovirus inoculum dose on virus kinetics, shedding, and symptoms. A) Fitted curves showing the full infection time-course. Onset is time at which virus load rose to the LOD1 level. Duration is amount of time where virus load was above the LOD1 level. Peak is time to virus peak shedding. B) Zoomed in plot of the first 7 days to better show the initial increase and peak. Curves and shaded regions indicate means and credible intervals of the fitted time series Bayesian model. LOD1 and LOD2 lines indicate the 2 different limits of detection. Missing values attributable to limits of detection were treated as censors (Appendix). AUC, area under virus concentration curve; GEC, genomic equivalent copies; LOD, limit of detection; RT-PCR, reverse transcription PCR.

We calculated the population-level curves per dose group for the estimated virus load trajectories (Figure 2) and developed fitted curves for each person (Appendix). The curves show a trend toward more rapid onset and earlier virus peak as dose increases (Figure 2, panel B) but little effect on shedding duration and total viral load (Figure 2, panel A). To further quantify these results, we sampled trajectories from the posterior distributions. For each trajectory, we computed 4 quantities (indicated in Figure 2, panel A): shedding onset (i.e., time at which virus became detectable), time of peak virus shedding, duration of virus shedding, and the total amount of virus shed (computed as area under the curve). We then examined the distribution of each of these quantities.

**Figure 3:**
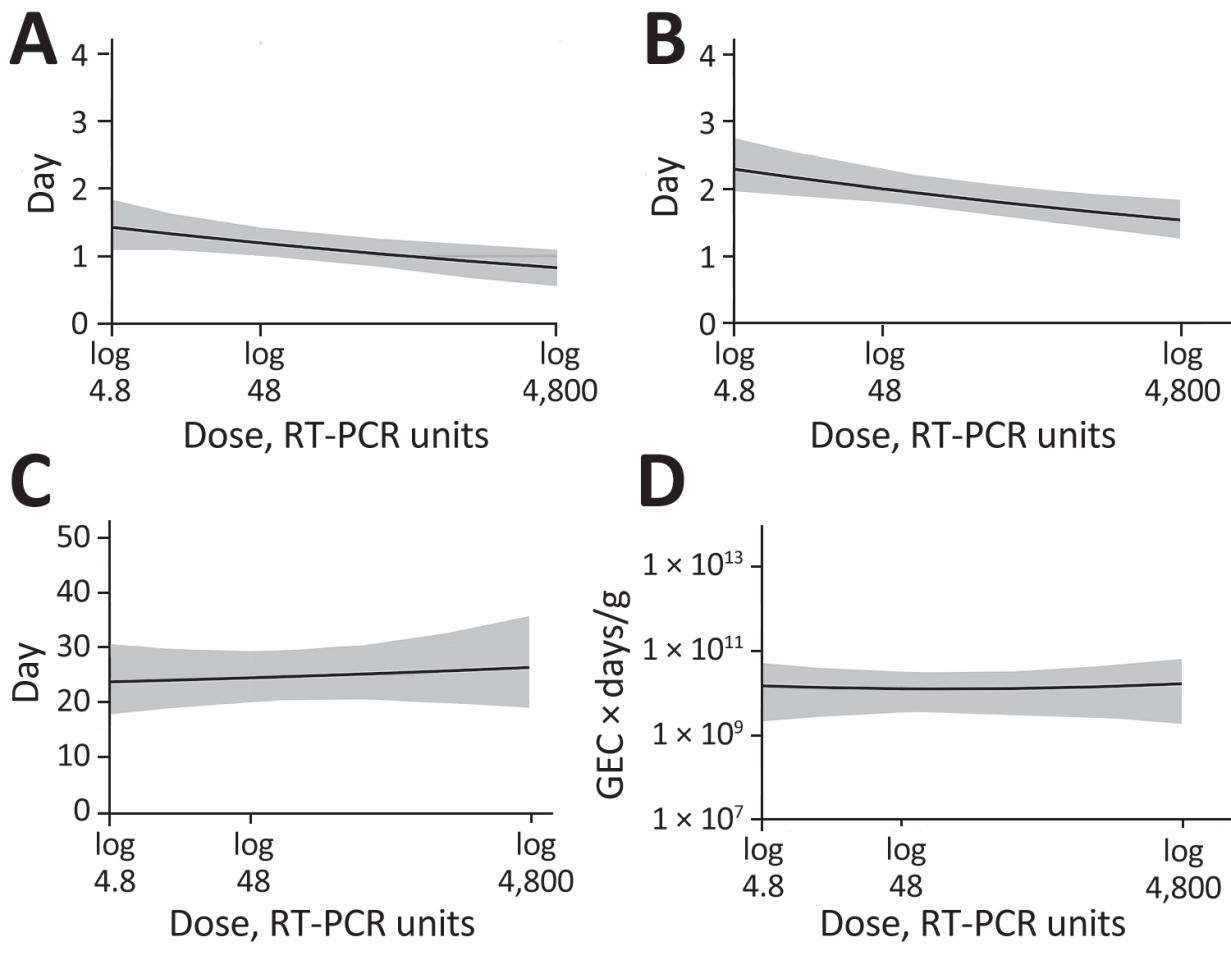
Associations between 4 characteristics of fecal viral shedding kinetics and levels of inoculum dose in patients in study of the effect of norovirus inoculum dose on virus kinetics, shedding, and symptoms. A) Shedding onset (time at which virus load reaches limit of detection 1). B) Time to virus peak shedding. C) Shedding duration (amount of time where virus load was above limit of detection 1). D) Total virus load (area under the fitted trajectory). Lines and shaded regions indicate means and 95% credible intervals of the posterior samples of the fitted time series model. RT-PCR, reverse transcription PCR.

We calculated the model-predicted relationship between dose and those 4 quantities (Figure 3). As the dose increased from 4.8 to 4,800 RT-PCR units, average onset time decreased from 1.4 (95% CrI 1.1–1.8) to 0.8 (95% CrI 0.5–1.1) days, and the time of virus peak decreased from 2.3 (95% CrI 2–2.8) to 1.5 (95% CrI 1.3–1.8) days. We observed a very slight trend toward increased duration of shedding, from 23.7 (95% CrI 17.8–30.6) to 26.4 (95% CrI 19–35.8) days. Total virus load barely changed, from 1.5 × 10^10^ (95% CrI 2.2 × 10^9^–5.2 × 10^10^) to 1.7 × 10^10^ (95% CrI 1.9 × 10^9^–6.6 × 10^10^) GEC × days/g.

### Association between Dose and Symptoms

**Figure 4:**
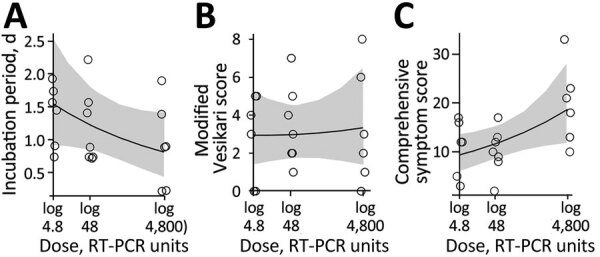
Association between inoculum dose and symptoms in patients in study of the effect of norovirus inoculum dose on virus kinetics, shedding, and symptoms. A) Incubation period (i.e., time between infection and onset of first symptoms). B) Severity using the modified Vesikari score. C) Severity using the comprehensive symptom score. Circles show raw data for participants. Lines and shaded regions indicate means and 95% credible intervals of the fitted Bayesian model. RT-PCR, reverse transcription PCR.

We investigated associations between dose and symptom related outcomes next. A higher inoculum dose was associated with a shorter incubation period (more rapid symptoms onset) (Figure 4). The incubation period decreased from 1.5 (95% CrI 0.9–2.5) to 0.8 (95% CrI 0.4–1.4) days as dose increased (Figure 4, panel A).

Our model estimated a slight increase in symptoms as measured by the MVS, from 2.9 (95% CrI 1.4–5.2) to 3.3 (95% CrI 1.4–6.5) as dose increased (Figure 4, panel B). The CSS showed a more pronounced increase, from 9.4 (95% CrI 6.1–13.6) to 18.7 (95% CrI 11.8–28.3) (Figure 4, panel C). A further analysis suggests that the different pattern seen for the MVS and CSS might be attributable to those symptoms that are part of the MVS not showing an association with dose, whereas a few symptoms (e.g., cramps, malaise, nausea) that are part of the CSS but not the MVS do show a correlation with dose (Appendix).

### Sensitivity Analyses

We performed 2 main sensitivity analyses (Appendix). In the first sensitivity analysis, we treated dose as categorical (low, medium, or high) instead of continuous. For this analysis, total virus shedding in feces and vomit was highest at the intermediate dose, though with overlap of the credible intervals for all doses. Similar to results for the main analysis, an increase in dose led to earlier onset and peak of shedding. Duration of shedding and total virus load concentration also suggested the highest levels at intermediate doses, although again with overlap in uncertainty estimates. Symptom onset was earlier, and the CSS measure increased, with no noticeable effect on the MVS measure.

In the second sensitivity analysis, we included 1 person infected after receiving the lowest dose (0.48 RT-PCR units). For this dataset, we found similar patterns of increasing total virus shedding in feces and vomit as dose increased. Also consistent with those results, onset and peak of shedding occurred earlier but duration of shedding and total virus load concentration did not change noticeably. Symptom onset was earlier and stronger based on the CSS measure, with no noticeable effect on the MVS.

The categorical analysis suggested similar patterns but supported, albeit very tentatively, that intermediate dose might be associated with the highest level of shedding. However, because only a single person fell into the lowest-dose category, a categorical analysis that included that person did not seem to be useful, so we did not perform such an analysis.

In time series models, we treated values below the limits of detection as censors. In other virus shedding models, we additionally performed 2 sensitivity analyses to explore the effect of choices for the values that are below the limits of detection. The conclusions remained consistent (Appendix).

## Discussion

We explored the effect of norovirus inoculum dose on infection and disease outcomes, an important gap in the literature. We found that an increased dose was associated with a faster onset and peak of virus shedding in feces (Figure 3, panel A, B) but not with fecal shedding duration and total virus concentration (Figure 3, panel C, D). A trend toward increased total shedding was noted for both feces and vomit (Figure 1). Our analysis also showed a pattern of accelerated onset of symptoms. Symptom severity showed an increase with inoculum dose for the CSS measure but not the MVS measure (Figure 4), possibly because only some symptoms are affected by dose, and those symptoms are captured by CSS but not MVS (Appendix). An increase in symptoms despite no noticeable change in virus load suggests that symptoms are mostly immune-mediated. We found mild evidence that a high virus growth rate associated with increased symptoms (Appendix); thus, a more rapid initial virus growth might trigger a stronger immune response. This finding could be tested in studies that measure components of the ensuing immune response.

Findings similar to ours have been reported for other enteric pathogens. The clinical manifestation of typhoid illness appears to be independent of inoculum dose, whereas the onset of symptoms was more rapid after a higher infectious dose (*34*). More rapid onset of symptoms after a larger infectious dose has also been observed with cholera infections (*35*). This finding could suggest a general pattern of dose-dependent incubation periods for enteric diseases. We did not find evidence of presymptomatic virus shedding, which could be attributable to the fact that diarrhea and vomit were considered as symptoms in our research, which explains the similar time of virus shedding onset time and incubation period.

The association between dose and severity might partially explain the results of several recent norovirus vaccine candidates. Those vaccines have shown limited effectiveness at reducing the risk for infection but do seem to reduce disease outcomes (*5*,*36*). Perhaps protection induced by current vaccine candidates (assumed to be mainly mediated by antibodies) is not enough to provide sterilizing immunity and thus prevent infection but can reduce the effective dose that starts an infection and thereby reduce symptoms. This pattern would be consistent with our findings here.

However, it is unclear what the typical norovirus dose is for natural infections and how that dose compares with the doses chosen in the challenge study data we analyzed. This uncertainty limits any possibility to generalize results obtained from challenge studies to natural infections or the potential role of vaccine candidates at influencing the effective inoculum size that starts an infection. Thus, potential clinical or epidemiologic implications of changes in dose for natural infections will need to await further investigations to determine the potential applicability of challenge study results to such natural infection settings.

Our analysis was a secondary data analysis of a limited number of persons, which resulted in wide credible intervals and constrained further explorations of nonlinear models. The associations we found may not equal to causality. As such, our results should be considered exploratory and need to be confirmed in future studies. Further studies, ideally with larger sample sizes, are needed. Larger sample sizes might also allow for stratification on the basis of host characteristics, which could yield information regarding possible interactions between host characteristics and dose–outcome relationships.

In conclusion, if we can assume that the associations we found have an underlying causal relation (something that needs to be confirmed in future studies), our results suggest that norovirus dose might affect some infection outcomes while not influencing others. Thus, when comparing results across challenge studies or trying to combine data from multiple studies for analysis, some care must be taken if doses are different. In some instances, combining data across studies seems reasonable, such as combining data from multiple studies to focus on viral shedding. However, for symptom-related outcomes and quantities that focus on norovirus infection kinetics, dose differences might lead to differences between studies that prohibit easy comparison. 

Appendix Additional information about effect of norovirus inoculum dose on virus kinetics, shedding, and symptoms.
