# Supplementary material for: Effect of Norovirus Inoculum Dose on Virus Kinetics, Shedding, and Symptoms
Source: Emerg Infect Dis. 2023 Jul;29(7):1349–56. doi: 10.3201/eid2907.230117 (PMC10310361; doi:10.3201/eid2907.230117)
Supplement: Appendix — Additional information about effect of norovirus inoculum dose on virus kinetics, shedding, and symptoms. [file 23-0117-Techapp-s1.pdf]

Article DOI: https://doi.org/10.3201/eid2907.230117

Effect of Norovirus Inoculum Dose on Virus Kinetics, Shedding, and Symptoms

Appendix

Overview

This document contains detailed model description, additional results, and instruction in reproducibility. We also provided a separate folder contains all materials for reproducibility (https://github.com/yangepigroup/eid230117).

Code and data files to reproduce results

Main files

The 1_manuscript folder contains the RMarkdown file of the main manuscript (manuscript_main_text.rmd) and auxiliary files (the template.docx help set font size, font family and so on). The 2_supplementary_materials folder contains supplementary_text.rmd (the RMarkdown file of the supplement, which produces this document), a data folder, which contains the data (norodata.rds), an analyses folder, which contains all R codes for data analysis, and a common_functions folder, which contains several functions used by the scripts in the the analyses folder.

Data file

The norodata.Rds (R data file (Rds)) contains a list with nine elements (Appendix Table 1). Each element contains parts of the data formatted in a way that is suitable for its intended analysis. These datasets were used by the R scripts in the analyses folder.

Data fvshed_gmu_96h_LOD12_0_15000 and fvshed_gmu_96h_LOD12_0_4e7 related to sensitivity analyses of limit detection issues that described in later sections.

The variables show in Appendix Table 2 are shared across all data subsets.

The dataset of ftsshedding (fecal virus shedding) has several variables necessary for later time-series modeling (Appendix Table 3).

The dataset of css_10symptoms has ten kinds of symptoms collected from the trial. The dataset of css has a variable (css) for comprehensive symptom score (CSS). The dataset of mvs has one variable (mvs) for the Modified Vesikari score (MVS), and five variables (mvsc1 to mvsc5) for five score components, respectively.

Analysis files

The analyses folder contains all R code for data analysis. For different outcomes, there is always one script that performs the model fitting (<OUTCOME>.R), and one for generating figures (<OUTCOME>_plot.R). Readers should choose an outcome and run the modeling code first, and then execute the plotting code. These are the existing scripts for fitting the different outcomes:

- allshedding_con.R fits models to the 3 total shedding outcomes, treating dose as continuous. The code fits models for both the 19 individuals shown in the main text, and the 20 individuals shown in the supplementary results.
- allshedding_cat.R fits models to the 3 total shedding outcomes, treating dose as categorical.
- tsshedding_con.R and tsshedding_cat.R fit the time-series data for fecal shedding to models, treating dose either continuous or categorical, respectively.

allsymptoms_con.R and allsymptoms_cat.R fit the incubation period, CSS, and MVS outcomes for continuous or categorical dose, respectively.

Model results are saved in the /results/rds/, and figures are saved in the /results/plots/.

Reproducing results

To reproduce all our analyses, please follow these steps:

1. Double click norovirus.Rproj.
2. Install necessary packages, including knitr, dplyr, tidyr, here, brms, huxtable, ggplot2, patchwork, stringr, ggthemes, ggpubr.
3. Open and run the R script allshedding_con.R, and then allshedding_con_plot.R.

4. Open and run the R script allsymptoms_con.R, and then allsymptoms_con_plot.R.
5. Open and run the R script allshedding_cat.R, and then allshedding_cat_plot.R.
6. Open and run the R script allsymptoms_cat.R, and then allsymptoms_cat_plot.R.
7. Open and run the R script tsshedding_con.R, and then tsshedding_con_plot.R.
8. Open and run the R script tsshedding_cat.R, and then tsshedding_cat_plot.R.
9. Open and run the RMD script manuscript_main_text.rmd, and supplementary_text.rmd.

In our Bayesian models, we implemented four chains with four CPU cores. Depending on the computer performance, models may need a few hours to run.

Analysis with rethinking package

All results presented in the main text and supplement are fit using Stan through the brms R package. As a check, we also implemented the same models with the rethinking package, which provides another R interface to the Stan fitting software.

The code for the models implemented with rethinking is in the folder 2_supplementary_materials/analyses/rethinking-analysis folder.

To run these scripts, the rethinking R package will need to be installed follwing the instructions here: *https://github.com/rmcelreath/rethinking*

The folder contains three scripts which must be run in the following order:

1. Open and run the R script models.R. This script fits the models to the data and consequently will have a long run time.
2. Open and run the R script preds.R. This script processes the Stan model objects and generates the model predictions of interest. Some steps, particularly processing the time series models, may have a long run time.
3. Open and run the R script figs.R. This script generates replicate figures similar to those included in the manuscript and the supplement.

All of the results (model objects, predictions, and figures) will be saved in the folder 2_supplementary_materials/analyses/rethinking-results. If this folder and the necessary

subfolders do not exist, they will be created for you the first time the script models.R is run. Finally, we recommend restarting the R session between scripts to ensure that sufficient memory is available.

Session information

We provide our PC session information as below:

```
## R version 4.2.3 (2023-03-15 ucrt)
## Platform: x86_64-w64-mingw32/x64 (64-bit)
## Running under: Windows 10 x64 (build 19045)
##
## Matrix products: default
##
## locale:
## [1] LC_COLLATE = English_United States.utf8
## [2] LC_CTYPE = English_United States.utf8
## [3] LC_MONETARY = English_United States.utf8
## [4] LC_NUMERIC = C
## [5] LC_TIME = English_United States.utf8
##
## attached base packages:
## [1] parallel stats graphics grDevices utils datasets methods
## [8] base
##
## other attached packages:
## [1] ggpubr_0.6.0 rethinking_2.31 cmdstanr_0.5.3
## [4] rstan_2.26.21 StanHeaders_2.26.21 ggthemes_4.2.4
## [7] stringr_1.5.0 patchwork_1.1.2 ggplot2_3.4.2
## [10] ftExtra_0.5.0 huxtable_5.5.2 brms_2.19.0
## [13] Rcpp_1.0.10 here_1.0.1 tidyr_1.3.0
## [16] dplyr_1.1.1 knitr_1.42
##
## loaded via a namespace [and not attached]:
```

```
## [1] uuid_1.1-0 backports_1.4.1 systemfonts_1.0.4
## [4] plyr_1.8.8 igraph_1.4.1 crosstalk_1.2.0
## [7] rstantools_2.3.1 inline_0.3.19 digest_0.6.31
## [10] htmltools_0.5.5 fansi_1.0.4 magrittr_2.0.3
## [13] checkmate_2.1.0 RcppParallel_5.1.7 matrixStats_0.63.0
## [16] officer_0.6.2 xts_0.13.0 askpass_1.1
## [19] gfonts_0.2.0 prettyunits_1.1.1 colorspace_2.1-0
## [22] textshaping_0.3.6 xfun_0.38 callr_3.7.3
## [25] crayon_1.5.2 jsonlite_1.8.4 zoo_1.8-11
## [28] glue_1.6.2 gtable_0.3.3 V8_4.2.2
## [31] distributional_0.3.2 car_3.1-2 pkgbuild_1.4.0
## [34] shape_1.4.6 abind_1.4-5 scales_1.2.1
## [37] fontquiver_0.2.1 mvtnorm_1.1-3 rstatix_0.7.2
## [40] miniUI_0.1.1.1 xtable_1.8-4 stats4_4.2.3
## [43] fontLiberation_0.1.0 DT_0.27 htmlwidgets_1.6.2
## [46] threejs_0.3.3 posterior_1.4.1 ellipsis_0.3.2
## [49] pkgconfig_2.0.3 loo_2.6.0 farver_2.1.1
## [52] utf8_1.2.3 crul_1.3 tidyselect_1.2.0
## [55] rlang_1.1.0 reshape2_1.4.4 later_1.3.0
## [58] munsell_0.5.0 tools_4.2.3 cli_3.6.1
## [61] generics_0.1.3 broom_1.0.4 evaluate_0.20
## [64] fastmap_1.1.1 yaml_2.3.7 ragg_1.2.5
## [67] processx_3.8.0 zip_2.2.2 purrr_1.0.1
## [70] nlme_3.1-162 mime_0.12 xml2_1.3.3
## [73] compiler_4.2.3 bayesplot_1.10.0 shinythemes_1.2.0
## [76] rstudioapi_0.14 curl_5.0.0 ggsignif_0.6.4
## [79] tibble_3.2.1 stringi_1.7.12 ps_1.7.4
## [82] Brobdingnag_1.2-9 gdtools_0.3.3 lattice_0.20-45
## [85] Matrix_1.5-3 fontBitstreamVera_0.1.1 markdown_1.5
## [88] shinyjs_2.1.0 tensorA_0.36.2 vctrs_0.6.1
## [91] pillar_1.9.0 lifecycle_1.0.3 bridgesampling_1.1-2
```

[94] data.table_1.14.8 flextable_0.9.1 httpuv_1.6.9
[97] R6_2.5.1 bookdown_0.33 promises_1.2.0.1
[100] gridExtra_2.3 codetools_0.2-19 colourpicker_1.2.0
[103] MASS_7.3-58.2 gtools_3.9.4 assertthat_0.2.1
[106] openssl_2.0.6 rprojroot_2.0.3 withr_2.5.0
[109] httpcode_0.3.0 shinystan_2.6.0 grid_4.2.3
[112] coda_0.19-4 rmarkdown_2.21 carData_3.0-5
[115] shiny_1.7.4 base64enc_0.1-3 dygraphs_1.1.1.6

Analysis of virus shedding

The following sections provide additional details and further results related to our analyses of virus shedding.

Accounting for limits of detection in virus shedding

The study that produced the data used two different methods to determine virus concentration in feces (*1*). The first method was a quantitative real-time RT-PCR (qRT-PCR) method. This method was able to detect virus up to a limit of detection of 40×10^6 genomic equivalent copies (GEC). If a sample was below this level (negative qPCR reading), the sample was retested using an immunomagnetic capture (IMC) RT-PCR assay. IMC RT-PCR is more sensitive, with a lower limit of detection of 15×10^3 GEC, but only produces a qualitative positive or negative readout. We labeled the limit of 15×10^3 GEC as LOD1, and 40×10^6 as LOD2. Thus, virus samples have a numeric value if they are above LOD2, are labeled as positive if they are below LOD2 but above LOD1, and are labeled as negative if they are below LOD1.

We dealt with these detection limits as follows.

For the analyses involving the time-series data of fecal virus shedding in our Bayesian nonlinear mixed effects model, we used the built-in approach of brms to handle censored values and deal with the limits of detection (*2*). In the time series models, the missing data were considered as censors. For example, with two LODs, 1) If the data was below both LODs (-,-), the interval was between 1 and 15000 (which leads to 0 and log(15000) on the modeling scale). 2) If the data was (+,-), the interval was between 15000 and 4e7; 3) If the data was (+,+), instead

of interval, the observed value was used. In brms, the likelihood function uses a cumulative distribution function for censored data (*3*).

For the simple analysis that does not use the time-series model, we substituted values. If a sample had a quantitative concentration above 40×10^6 GEC, we used the numeric value. If a sample was reported as positive (concentration between 40×10^6 GEC and 15×10^3 GEC), we used the geometric mean of those two values ($\approx 7.75 \times 10^5$ GEC). Similarly, if a sample was recorded as negative (concentration below 15×10^3 GEC), we used the geometric mean of 15×10^3 GEC and 1 GEC (≈ 122 GEC). To explore the impact of these choices, we additional performed two sensitivity analyses. We set values below the LOD1 to 1, and values between LOD1 and LOD2 were set to either 15000 (the LOD1 value) or 4e7 (the LOD2 value), to explore two extremes. These additional analyses are shown further below in this supplement.

To compute the total amount of virus shed per shedding event, we multiplied the virus concentration with the weight of the shed feces (i.e., GEC/g × weight of feces). Finally, for each individual, we summed values for all shedding events.

Data for vomiting had similar limits of detection. These data were recorded as either a numeric value above 2,200 GEC, a positive readout below $2{,}200$ GEC or a negative readout. For the vomiting data, we did not have information on the limit of detection for the qualitative assay. Based on a comparison of the two methods (*4*), we made the assumption that the LOD for the qualitative assay was a factor of 10 lower, thus 220 GEC. We then again took the geometric mean of 2,200 GEC and 220 GEC for the positive values (≈ 696 GEC), and 220 GEC and 1 GEC for the negative values (≈ 15 GEC). All virus concentrations were then multiplied with total vomit volume (i.e., virus particles/ml × volume of vomit), then summed those for each individual. This included making the additional assumption that 1 g of vomit equated to 1 mL (*5*).

Modeling total virus shedding

We computed total virus shedding by multiplying virus concentration with sample weight (feces) or volume (vomit) for each shedding event, and summing all values for each individual.

We applied a mixed effects model that investigated associations between the outcomes (total shedding amount) and inoculum dose. For the analysis shown in the main text, we assumed a linear relationship between the log of the dose (x_i) and the log of the outcome (y_i).

We fitted the same model to each of the 3 outcomes. Those are 1) fecal shedding during the 96 hours under clinical observation, 2) fecal shedding including the time at which individuals recorded shedding events at home, and 3) vomit shedding (which occurred only during the first 96 hours).

The mathematical definition of the model is as follows:

Likelihood:

$$y_i \sim \text{Normal}(\mu_i, \sigma)$$

Linear model:

$$\mu_i = \alpha_i + \beta(x_i - x^*)$$

Adaptive priors:

$$\alpha_i \sim \text{Normal}(\delta, \gamma)$$

Fixed priors:

$$\delta \sim \text{Normal}(25,5)$$
$$\gamma \sim \text{Half-Cauchy}(0,2)$$
$$\beta \sim \text{Normal}(0,1)$$
$$\sigma \sim \text{Half-Cauchy}(0,2)$$

The outcome of interest, y_i, is the total amount of virus shedding (in log units) of individual i. We assumed this value to be normally distributed with mean, μ_i, and an overall standard deviation, σ. The mean was assumed to have an individual-specific component and a dose-dependent component. The parameter α_i describes the individual-level, dose-independent expected mean of the outcome for each individual, the parameter β encodes the potential impact of the dose, x_i, that the individual i received. To make priors easier to define and interpret, we subtracted the intermediate dose from the dose values, i.e., we set $x^* = \log(48)$. This adjustment implies that the parameter α_i represents the expected total amount of virus shedding when the inoculum dose is at this intermediate value. (Without subtraction of x^*, α_i would represent total shedding if the dose was zero, which is biologically not meaningful and makes assigning reasonable priors more difficult (*6*).)

Priors are chosen based on what we know about the virus kinetics, and to ensure prior predictive simulations produce flexible but reasonable outcomes (*6*). We used Half-Cauchy

distributions for priors of standard deviation, because Cauchy has fatter tails than normal distribution which will make priors less informative. Since vomit outcome values were lower than those for feces, we adjusted the α_i prior for this model to have a mean around 20.

As part of our sensitivity analyses, presented below, we also explored a model which treats dose as categorical. The following lines in the model are adjusted accordingly:

$$
\begin{aligned}
&\text{Linear model:} \\
&\qquad \mu_i = \alpha_i + \beta_{dose_i} \\
&\text{Adaptive priors:} \\
&\qquad \alpha_i \sim \text{Normal}(0, \gamma) \\
&\text{Fixed priors:} \\
&\qquad \gamma \sim \text{Half-Cauchy}(0,2) \\
&\qquad \beta_{dose_i} \sim \text{Normal}(25,5),
\end{aligned}
$$

In this notation, the parameter β_{dose_i} takes on discrete values based on the dose level of each individual, i. Specifically, the 3 categories are low, medium, or high dose, corresponding to 4.8, 48, or 4800 RT-PCR units.

Modeling longitudinal virus concentration kinetics

To model the longitudinal time-series of virus concentration in feces, we used a previously developed equation that was shown to describe virus time-course in acute viral infections well (*7*). This equation provides a good empirical function to fit the increase, then decrease of viral load seen in many acute viral infections. The equation has four parameters and is given by:

$$V(t) = \frac{2p}{e^{-g(t-T)} + e^{d(t-T)}}$$

The outcome of interest is virus load as a function of time, $V(t)$. The model parameters approximately represent the peak virus load (p), the initial exponential growth rate (g), the time of virus peak (T) and the eventual rate of virus decline (d). (The parameters only approximately map to those biologic quantities, see (*7*) for details.)

Since all parameters in the model above need to be positive to achieve biologically reasonable trajectories for $V(t)$, we rewrote the equation for our purpose with exponentiated parameters. That is, we assume that the deterministic portion of the virus load data (on a log scale, denoted below as $\mu_{i,t}$), is described for each individual, i, by

$$\mu_{i,t} = \log\left(\frac{2\exp(p_i)}{e^{-\exp(g_i)(t_i - \exp(T_i))} + e^{\exp(d_i)(t_i - \exp(T_i))}}\right)$$

Since there was a fair amount of variability in the virus load data, we modeled the outcome using a non-standardized Student-t distribution instead of a Normal distribution, which provides more robust estimates in the presence of strong variability (*2*,*6*). Specifically, we model the virus load data as

$$y_{i,t} \sim \text{Student-t}(k, \mu_{i,t}, \sigma)$$

where the standard deviation (σ) is modeled with a Half-Cauchy distribution, and the degrees of freedom (k) are modeled with a Gamma distribution with priors as shown in the equations below. We assumed that each of the four model parameters of the $\mu_{i,t}$ equation follow normal distributions, and can be described by linear models, each with an individual-level intercept parameter and a parameter quantifying the potential impact of dose. We again modeled the latter for our main analysis as being a linear function of the log of the dose. As described above, we again subtracted the intermediate dose to make the intercept parameters biologically meaningful. The model equations are given by:

Likelihood:

$$y_{i,t} \sim \text{Student}\left(k, \mu_{i,t}, \sigma\right)$$

Overall time-series equation:

$$\mu_{i,t} = \log\left(\frac{2\exp(p_i)}{e^{-\exp(g_i)\left(t_i - \exp(T_i)\right)} + e^{\exp(d_i)\left(t_i - \exp(T_i)\right)}}\right)$$

Parameter equations:

$$p_i = p_{0,i} + p_1 \cdot (x_i - x^*)$$
$$g_i = g_{0,i} + g_1 \cdot (x_i - x^*)$$
$$T_i = T_{0,i} + T_1 \cdot (x_i - x^*)$$
$$d_i = d_{0,i} + d_1 \cdot (x_i - x^*)$$

Adaptive priors:

$$p_{0,i} \sim \text{Normal}\left(\mu_p, \sigma_p\right)$$
$$g_{0,i} \sim \text{Normal}\left(\mu_g, \sigma_g\right)$$
$$T_{0,i} \sim \text{Normal}(\mu_T, \sigma_T)$$
$$d_{0,i} \sim \text{Normal}(\mu_d, \sigma_d)$$

Fixed priors:

$$\mu_p \sim \text{Normal}(25,5)$$
$$\sigma_p \sim \text{Half-Cauchy}(0,1)$$
$$\mu_g \sim \text{Normal}(3,1)$$
$$\sigma_g \sim \text{Half-Cauchy}(0,1)$$
$$\mu_T \sim \text{Normal}(0,1)$$
$$\sigma_T \sim \text{Half-Cauchy}(0,1)$$
$$\mu_d \sim \text{Normal}(-1,0.5)$$
$$\sigma_d \sim \text{Half-Cauchy}(0,1)$$
$$p_1 \sim \text{Normal}(0,1)$$
$$g_1 \sim \text{Normal}(0,0.5)$$
$$T_1 \sim \text{Normal}(0,0.5)$$
$$d_1 \sim \text{Normal}(0,0.5)$$
$$k \sim \text{Gamma}(shape = 2, rate = 0.1)$$
$$\sigma \sim \text{Half-Cauchy}(0,1)$$

Values for priors were chosen to be weakly informative, such that prior predictive simulations produced virus-load trajectories that made biologic sense, while still allowing for a wide variety of possible trajectories to be informed by fitting to the data. The peak level of virus concentration for norovirus in our data and previous studies is broadly in the range of 10^{10} to 10^{14} (*1*,*8*,*9*) (around 23–35 in log units). We approximately centered our prior around those values, while allowing for a broad standard deviation so that the data will dominate the posterior results. Similarly, the growth rate, μ_g, will have a value such that the peak is reached within the

first several days following infection. A Normal distribution with a mean of 3 (in log units) can produce the necessary range of value. Virus is expected to peak a few days following infection. Thus we chose μ_T and T_1 to have normal distributions with a spread such that the range of values for $\exp(T)$ is centered around the first few days. The decay rate, μ_d, needs to allow for a decline of virus to undetectable levels that can be as fast as a week or longer than a month. A Normal distribution with log-transformed mean of -1 can produce such outcomes.

We performed prior predictive simulations to ensure that our priors led to biologically reasonable time-series trajectories, while also being broad and flexible enough to let the data dominate the posterior distribution.

For the sensitivity analysis, where we treated dose as a categorical variable, the dose-associated parameters change and now are assigned distinct values based on dose category. The following components of the above model changed, with the rest remaining the same:

Parameter equations:

$$
\begin{aligned}
p_i &= p_{0,i} + p_{1,dose_i} \\
g_i &= g_{0,i} + g_{1,dose_i} \\
T_i &= T_{0,i} + T_{1,dose_i} \\
d_i &= d_{0,i} + d_{1,dose_i}
\end{aligned}
$$

Adaptive priors:

$$
\begin{aligned}
p_{0,i} &\sim \text{Cauchy}(0, \gamma_p) \\
p_{0,i} &\sim \text{Cauchy}(0, \gamma_g) \\
p_{0,i} &\sim \text{Cauchy}(0, \gamma_T) \\
p_{0,i} &\sim \text{Cauchy}(0, \gamma_d)
\end{aligned}
$$

Fixed priors:

$$
\begin{aligned}
\gamma_p &\sim \text{Half-Cauchy}(0,1) \\
\gamma_g &\sim \text{Half-Cauchy}(0,1) \\
\gamma_T &\sim \text{Half-Cauchy}(0,1) \\
\gamma_d &\sim \text{Half-Cauchy}(0,1) \\
p_{1,dose_i} &\sim \text{Normal}(25,5) \\
g_{1,dose_i} &\sim \text{Normal}(3,1) \\
T_{1,dose_i} &\sim \text{Normal}(0,1) \\
d_{1,dose_i} &\sim \text{Normal}(-1,0.5)
\end{aligned}
$$

As before, in this categorical analysis, x_i is the dose category for individual i, and is either low, medium or high.

Analysis of symptom outcomes

The following sections provide additional details and further results related to our analyses of infection symptoms.

We considered three different symptom-related outcomes, namely time to first symptom onset (incubation period) and two versions of scores that quantify overall infection severity.

Since individuals were allowed to leave the study center after 96 hours and illness is typically self-limiting lasting for only a few days (*10*), we calculated the scores based on records in the first 4 days.

Incubation period

Incubation period, i.e., the time between infection and onset of symptoms, was directly computed from the data as the time-span between reported time at which the infection challenge was administered, and the time at which the first symptom was reported.

Modified Vesikari score (MVS)

The modified Vesikari score (MVS) is a previously defined quantity that has been used in a modified form by several studies to measure norovirus severity (*11–14*). The score has the seven components shown in Appendix Table 4. For our dataset, since volunteers were housed in a healthcare setting, the health care provider visit component was not applicable, and we thus removed it from the score calculation (*13*). Since we did not have information on treatment, we also dropped that component. This left us with a five-component score, which was computed for each individual following the rules shown in Appendix Table 4.

Appendix Table 5 shows the scores for all 20 volunteers in the study that were part of our analyses.

Comprehensive symptom score (CSS)

In addition to the modified Vesikari score, we defined and computed a comprehensive symptom score which took all recorded symptoms into account.

The study reported the following symptoms: body temperature, malaise, muscle aches, headache, nausea, chills, anorexia, cramps, unformed or liquid feces, and vomiting. Clinical symptoms (except feces and vomiting) were reported as none, mild, moderate, or severe, which

we coded as a score of 0 to 3. For feces, we used a scoring of solid = 0, unformed = 1, and liquid = 2. Vomit was reported as absent or present and scored as 0 or 1.

Individuals had their symptoms recorded at different times and frequencies throughout the day. Thus, summing up the recorded scores would have introduced bias due to different recording frequencies. Thus, we instead determined the highest score per symptom for each individual per day, and summed those. This produced a daily total symptom score for each individual. We then summed those to obtain our comprehensive symptom score. For example, if one individual had daily total symptom score values of 5 (1st day), 10 (2nd day), 2 (3rd day), and 0 (4th day), the final total symptom scores would be 17.

Appendix Table 6 shows the daily and total comprehensive score values for the 20 individuals, Appendix Figure 1 shows the same information in graphical form, stratified by dose.

Modeling symptom outcomes

Using following models, we tested the impact of dose on each kind of symptom in the trial and two types of symptom scores.

The incubation period is positive and not too far from zero (as measured in days). Thus, to prevent any possible negative outcomes, we assumed it followed a log-normal distribution. The rest of the model is similar to those implemented for the total shedding outcomes, with the full model is given below.

$$\begin{aligned}
&\text{Likelihood:} \\
&Y_i \sim \text{Log-Normal}(\mu_i, \sigma) \\
&\text{Linear model:} \\
&\mu_i = \alpha_i + \beta(x_i - x^*) \\
\text{Adaptive priors:}& \\
&\alpha_i \sim \text{Normal}(\delta, \gamma) \\
\text{Fixed priors:}& \\
&\delta \sim \text{Normal}(1,5) \\
&\gamma \sim \text{Half-Cauchy}(0,2) \\
&\beta \sim \text{Normal}(0,1) \\
&\sigma \sim \text{Half-Cauchy}(0,2),
\end{aligned}$$

As we did for the total shedding outcomes, the model was adjusted for the categorical dose sensitivity analysis as follows.

Linear model:
$$\mu_i = \alpha_i + \beta_{dose_i}$$

Adaptive priors:

α prior: $$\alpha_i \sim \text{Normal}(0, \gamma)$$

Fixed priors:

$$\gamma \sim \text{Half-Cauchy}(0,2)$$
$$\beta_{dose_i} \sim \text{Normal}(1,5)$$

We used the same overall model structure for the MSV and CSS outcomes. However, we modeled these outcomes using a Gamma-Poisson distribution (also called negative binomial distribution) for the likelihood, since both scores are nonnegative integer-valued. The Gamma-Poisson model allows for additional variance (also called overdispersion) compared to a Poisson distribution. The variance is determined by the ϕ parameter, and the rate λ is similar to the rate of an ordinary Poisson distribution. We use the customary log-link for λ, and model its functional relationship with dose as above with an individual-level intercept and a dose-specific component, linearly dependent on the log of the dose. The model is given by the following set of equations.

Likelihood:
$$y_i \sim \text{Gamma-Poisson}(\lambda_i, \phi)$$

Linear model:
$$\log(\lambda_i) = \alpha_i + \beta(x_i - x^*)$$

Adaptive priors:
$$\alpha_i \sim \text{Normal}(\delta, \gamma)$$

Fixed priors:
$$\delta \sim \text{Normal}(1,5)$$
$$\gamma \sim \text{Half-Cauchy}(0,2)$$
$$\beta \sim \text{Normal}(0,1)$$
$$\phi \sim \text{Half-Cauchy}(0,2)$$

Values for the prior distributions were again chosen to ensure reasonable prior predictive results for the outcomes. To model dose as a categorial variable, we changed the following parts of the model.

$$\text{Linear model:}$$
$$\log(\lambda_i) = \alpha_i + \beta_{dose_i}$$

Adaptive priors:

$$\alpha_i \sim \text{Normal}(0, \gamma)$$

Fixed priors:

$$\gamma \sim \text{Half-Cauchy}(0,2)$$
$$\beta_{dose_i} \sim \text{Normal}(1,5)$$

Model setup, outcomes and diagnostics

In our Bayesian models, we generally implemented four chains with ≈30k iterations with 25k as a warmup to obtain 5k posterior samples for final results. We assessed Rhat, mixing of chains, and posterior distributions to ensure convergence.

As described above, the models were implemented in R using the brms package. We report results based on predictions from the posterior samples of the models (*3*). The predictions are based on an assumption that a new individual, who theoretically similar to individuals in this trial but has not been enrolled previously, be given with a certain amount of virus. Then, the model posterior predictive distribution of the new individual was summarized by a mean and credible interval for an outcome.

Evaluation of prior and posterior distributions for model parameters

To ensure that our results were not overly influenced by the choice of prior distributions, we compared prior and posterior distributions.

The parameter's prior and posterior distributions showed that the choice of prior distribution had no significant impact on our results (Appendix Figure 2 to 4).

Results from additional analyses

Fecal virus shedding during the full observation period

In the main text, we focus on the data collected during the time individuals were under direct observation, since this ensures complete collection of all feces. Here, we investigate the relation between dose and total fecal shedding for all samples, including those individuals were

asked to collect after returning home. We did not find evidence for associations between dose and virus shedding.

Impact of changing assumption for LOD values

As described above, for the non time-series analysis shown in the main text, we set measurements that were below LOD2 but above LOD1 to the geometric mean of those 2 values, and values below LOD1 were set to the geometric mean of LOD1 and 1. To explore the impact of these choices, we tested a few different choices for those values. The next figures show that the overall observed pattern remains consistent, independent of the choices for the LOD values.

Vomit events

In each dose group, only a few individuals had vomiting events. Some of these individuals had multiple vomiting events. Appendix Figure 8 graphically displays the vomiting event data. The recorded vomiting events were not sufficient to allow for a time-series analysis.

Individual-level fits to fecal sheedding time series data

The longitudinal virus shedding data in feces and fitted model results are shown in Appendix Figure 9 for each individual.

Correlation between total virus shed in feces and virus load

We compared the fitted total virus load (area under the curve), to the observed total virus shed in feces to assess model performance. We found strong correlation, which support the robustness of our models.

Assessing association of individual symptoms with dose

In the main text, we show that there is little association between dose and symptoms if quantified by the MVS, but there is an association if quantified by the CSS. To understand this difference, we explored correlations between all individual symptoms and dose. While the uncertainty intervals are wide, the figure below shows that symptoms that are part of the MVS (Feces form, Body temperature, and Vomit) show very little variation with dose, while a few other symptoms, which are part of CSS but not MVS, do show some dose variation (Cramps, Malaise and Nausea). This is likely the reason why CSS shows variation with dose, while MVS does not. A similar pattern is seen when dose is treated categorically, as shown below.

Assessing association of symptoms with virus growth rate

In the discussion, we speculate that the association of dose with increased symptoms might be mediated by a more rapid onset of the disease, due to more rapid initial virus growth at higher doses. To test this, we used the fitted individual level virus load curves and calculated the virus growth rate by

$$g_v = \frac{v_{t=0.625} - v_{t=0.5}}{0.625 - 0.5},$$

where, g_v is virus concentration growth rate; $v_{t=0.625}$ is the virus concentration value at day 0.625; $v_{t=0.5}$ is the time of virus concentration at day 0.5. We arbitrarily chose the two time points to make sure the virus concentration is in the exponential growth phase and has not yet started to level off.

The following figures show the association between growth rate and symptom scores (MVS and CSS) for each individual. They are nonlinear curves because negative binomial distributions were used in models similarly to the symptom scores (Appendix Figure 4, main manuscript). The range of x-axis was based on data.

Treating dose as a categorical variable

The following figures repeat the analyses and results shown in the main text, but now with dose treated as categorical.

Assessing association of total virus shedding with dose

Appendix Figure 14 shows data and model estimates for total shedding for each dose category, where low/medium/high indicate dose levels of 4.8, 48, and 4800 RT-PCR units. The overall pattern is similar to the one we found for the continuous analysis presented in the main text.

Modeling of virus concentration in feces

Appendix Figures 15 – 17 show data and model estimates for the longitudinal time-series analysis of the virus concentration in feces, with dose now treated as categorical (low, medium, or high). Again, the overall observed patterns are similar to the ones we found for the continuous analysis presented in the main text.

Assessing association of symptoms with dose

Appendix Figure 18 shows data and model estimates for the different symptom outcomes for each dose category. Again, the patterns seen are similar to those shown in the main text. One difference is that the comprehensive score only increases for the highest dose group, while the two lower groups are similar.

Looking at 95% credible interval of each symptom's score (horizontal bars in Appendix Figure 19) by 3 dose groups, we again find the pattern reported above for the case of treating dose as continuous. Namely, symptoms associated with MVS do not show any pronounced variation with dose, while several symptoms that are part of the CSS but not the MVS do show variation with dose.

Analyses with inclusion of the very-low dose infected individual

As explained in the main text, the original study also administered a dose of 0.48 RT-PCR units. At that dose level, only a single challenged individual became infected. We removed this person for the analysis presented in the main text. However, we also decided to conduct a sensitivity analysis that re-computes all results shown in the main text, now with the additional individual included.

Assessing association of total virus shedding with dose

Comparison of this figure with the one shown in the main text shows overall similar results.

Modeling of virus concentration in feces

As Appendix Figures 21 – 23 show, the findings remain essentially unchanged for these outcomes compared to what is shown in the main text.

Assessing association of symptoms with dose

As Appendix Figure 24 shows, the association between dose and symptom outcomes also remains essentially unchanged when including the additional individual in the analysis. We also again find a similar pattern between individual symptoms and dose as found previously (Appendix Figure 25).

References

1. Atmar RL, Opekun AR, Gilger MA, Estes MK, Crawford SE, Neill FH, et al. Norwalk virus shedding after experimental human infection. Emerg Infect Dis. 2008;14:1553–7. PubMed https://doi.org/10.3201/eid1410.080117

2. Bürkner P-C. Advanced Bayesian multilevel modeling with the R Package brms. R J. 2018;10:395. https://doi.org/10.32614/RJ-2018-017

3. Gelman A, Carlin JB, Stern HS, Dunson DB, Vehtari A, Rubin DB. Bayesian data analysis. 3rd edition. Boca Raton: CRC Press; 2013.

4. Gilpatrick SG, Schwab KJ, Estes MK, Atmar RL. Development of an immunomagnetic capture reverse transcription-PCR assay for the detection of Norwalk virus. J Virol Methods. 2000;90:69–78. PubMed https://doi.org/10.1016/S0166-0934(00)00220-2

5. Kirby AE, Streby A, Moe CL. Vomiting as a symptom and transmission risk in norovirus illness: evidence from human challenge studies. PLoS One. 2016;11:e0143759. PubMed https://doi.org/10.1371/journal.pone.0143759

6. McElreath R. Statistical rethinking: a Bayesian course with examples in R and Stan. 2nd edition. Boca Raton: Taylor and Francis, CRC Press; 2020.

7. Holder BP, Beauchemin CA. Exploring the effect of biological delays in kinetic models of influenza within a host or cell culture. BMC Public Health. 2011;11(Suppl 1):S10. PubMed https://doi.org/10.1186/1471-2458-11-S1-S10

8. Lee N, Chan MCW, Wong B, Choi KW, Sin W, Lui G, et al. Fecal viral concentration and diarrhea in norovirus gastroenteritis. Emerg Infect Dis. 2007;13:1399–401. PubMed https://doi.org/10.3201/eid1309.061535

9. Teunis PFM, Sukhrie FHA, Vennema H, Bogerman J, Beersma MFC, Koopmans MPG. Shedding of norovirus in symptomatic and asymptomatic infections. Epidemiol Infect. 2015;143:1710–7. **PMID 25336060**

10. Matthews JE, Dickey BW, Miller RD, Felzer JR, Dawson BP, Lee AS, et al. The epidemiology of published norovirus outbreaks: a review of risk factors associated with attack rate and genogroup. Epidemiol Infect. 2012;140:1161–72. PubMed https://doi.org/10.1017/S0950268812000234

11. Ruuska T, Vesikari T. Rotavirus disease in Finnish children: use of numerical scores for clinical severity of diarrhoeal episodes. Scand J Infect Dis. 1990;22:259–67. PubMed https://doi.org/10.3109/00365549009027046

12. Freedman SB, Eltorky M, Gorelick M; Pediatric Emergency Research Canada Gastroenteritis Study Group. Evaluation of a gastroenteritis severity score for use in outpatient settings. Pediatrics. 2010;125:e1278–85. PubMed https://doi.org/10.1542/peds.2009-3270

13. Atmar RL, Bernstein DI, Harro CD, Al-Ibrahim MS, Chen WH, Ferreira J, et al. Norovirus vaccine against experimental human Norwalk virus illness. N Engl J Med. 2011;365:2178–87. PubMed https://doi.org/10.1056/NEJMoa1101245

14. Bierhoff M, Arvelo W, Estevez A, Bryan J, McCracken JP, López MR, et al. Incidence and clinical profile of norovirus disease in Guatemala, 2008–2013. Clin Infect Dis. 2018;67:430–6. PubMed https://doi.org/10.1093/cid/ciy091

Appendix Table 1. Description of norodata.Rds

Files	Notes
data_tab1	Data description
feces96	Virus shedding in feces (96 h)
fvshed_gmu_96h_LOD12_0_15000	Virus shedding in feces (96 h) low limit for LODs
fvshed_gmu_96h_LOD12_0_4e7	Virus shedding in feces (96 h) up limit for LODs
fecesall	Virus shedding in feces (all)
vomit	Virus shedding in vomit (all)
ftsshedding	Time-series virus shedding in feces
vtsshedding	Time-series virus shedding in vomit
incubation	Onset of symptoms (Incubation)
css_10symptoms	Comprehensive symptom score components (CSSC)
css	Comprehensive symptom score (CSS)
mvs	Modified Vesikari score (MVS)

Appendix Table 2. Description of four variables

NO.	Variable	Notes
1	ID	Individual ID
2	dose	Amount of dose (RT-PCR unit)
3	dose_cat	Amount of dose (categorical version)
4	dose_log	Dose variable used in models (log(Amount of dose) - log(48))

Appendix Table 3. Description of variables necessary for later time-series modeling

NO.	Variable	Notes
1	y_censor	Indicator of censoring due to LOD, 0 = no censoring, 2 = interval censoring
2	y_virus	Observed virus in the feces
3	yc	Lower bound of the interval censoring
4	logy	Natural log of the observed virus in the feces
5	logyc	Natural log of the lower bound of the interval censoring

Appendix Table 4. Modified vesikari score components

Components	Score = 0	Score = 1	Score = 2	Score = 3
C1: Diarrhea duration, days	0	1 - 4	5	> = 6
C2: Maximum number of daily diarrheal stools	0	1 - 3	4 - 5	> = 6
C3: Vomiting duration, days	0	1	2	> = 3
C4: Maximum number of daily vomiting episodes	0	1	2 - 4	> = 5
C5: Maximum recorded fever	Not Elevated	Moderate	Mild	Severe
Health care provider visits	N/A	N/A	N/A	N/A
Treatment	N/A	N/A	N/A	N/A

Appendix Table 5. Modified vesikari scores for each individual.

ID	Dose	C1	C2	C3	C4	C5	Total
ID1	4800	0	0	1	2	0	3
ID2	4800	0	0	0	0	0	0
ID3	4800	0	0	0	0	1	1
ID4	4800	1	1	0	0	0	2
ID5	4800	1	2	1	2	2	8
ID6	4800	1	1	2	2	0	6
ID7	48	1	1	0	0	0	2
ID8	48	1	1	0	0	0	2
ID9	48	0	0	0	0	1	1
ID10	48	1	1	1	2	2	7
ID11	48	0	0	1	2	1	4
ID12	48	1	1	1	2	0	5
ID13	48	0	0	1	2	0	3
ID14	4.8	1	1	1	2	0	5
ID15	4.8	0	0	1	2	0	3
ID16	4.8	0	0	1	2	1	4
ID17	4.8	1	1	1	2	0	5
ID18	4.8	0	0	0	0	0	0
ID19	4.8	0	0	0	0	0	0
ID20	0.48	0	0	1	2	0	3

Appendix Table 6. Comprehensive symptom score values.

ID	Dose	Day1	Day2	Day3	Day4	Total
ID1	4800	1	7	2	0	10
ID2	4800	1	7	5	0	13
ID3	4800	0	6	13	2	21
ID4	4800	0	9	9	0	18
ID5	4800	1	14	15	3	33
ID6	4800	7	8	8	0	23
ID7	48	1	5	2	0	8
ID8	48	1	2	7	3	13
ID9	48	0	0	2	0	2
ID10	48	1	14	2	0	17
ID11	48	1	9	2	0	12
ID12	48	0	9	0	0	9
ID13	48	0	10	0	0	10
ID14	4.8	0	10	2	0	12
ID15	4.8	1	1	12	3	17
ID16	4.8	0	8	4	0	12
ID17	4.8	4	4	8	0	16
ID18	4.8	0	1	1	1	3
ID19	4.8	0	2	3	0	5
ID20	0.48	2	7	3	0	12

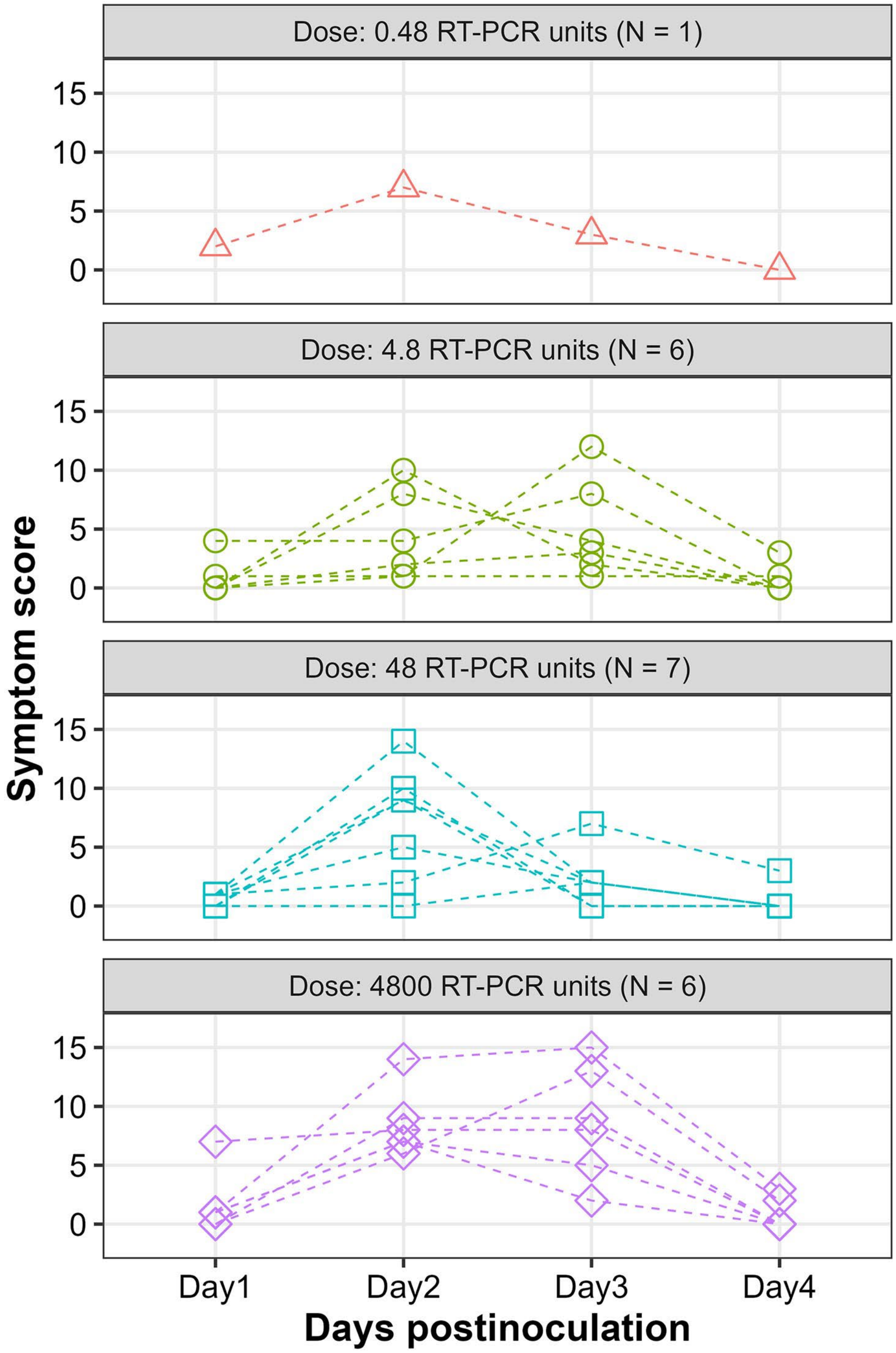

Appendix Figure 1. Daily comprehensive symptom score stratified by inoculum dose group

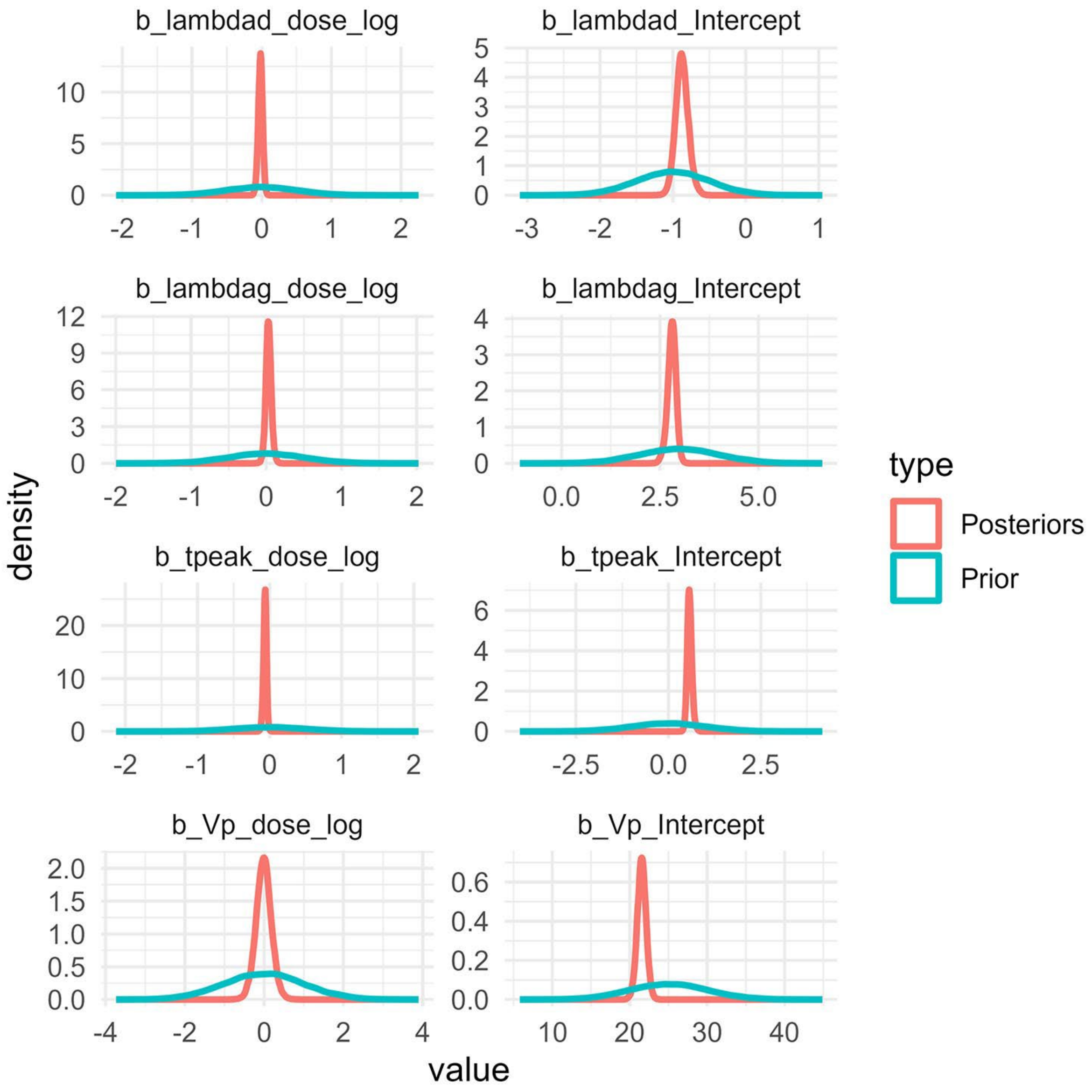

Appendix Figure 2. Comparisons of parameters' prior and posterior distributions for the main model.

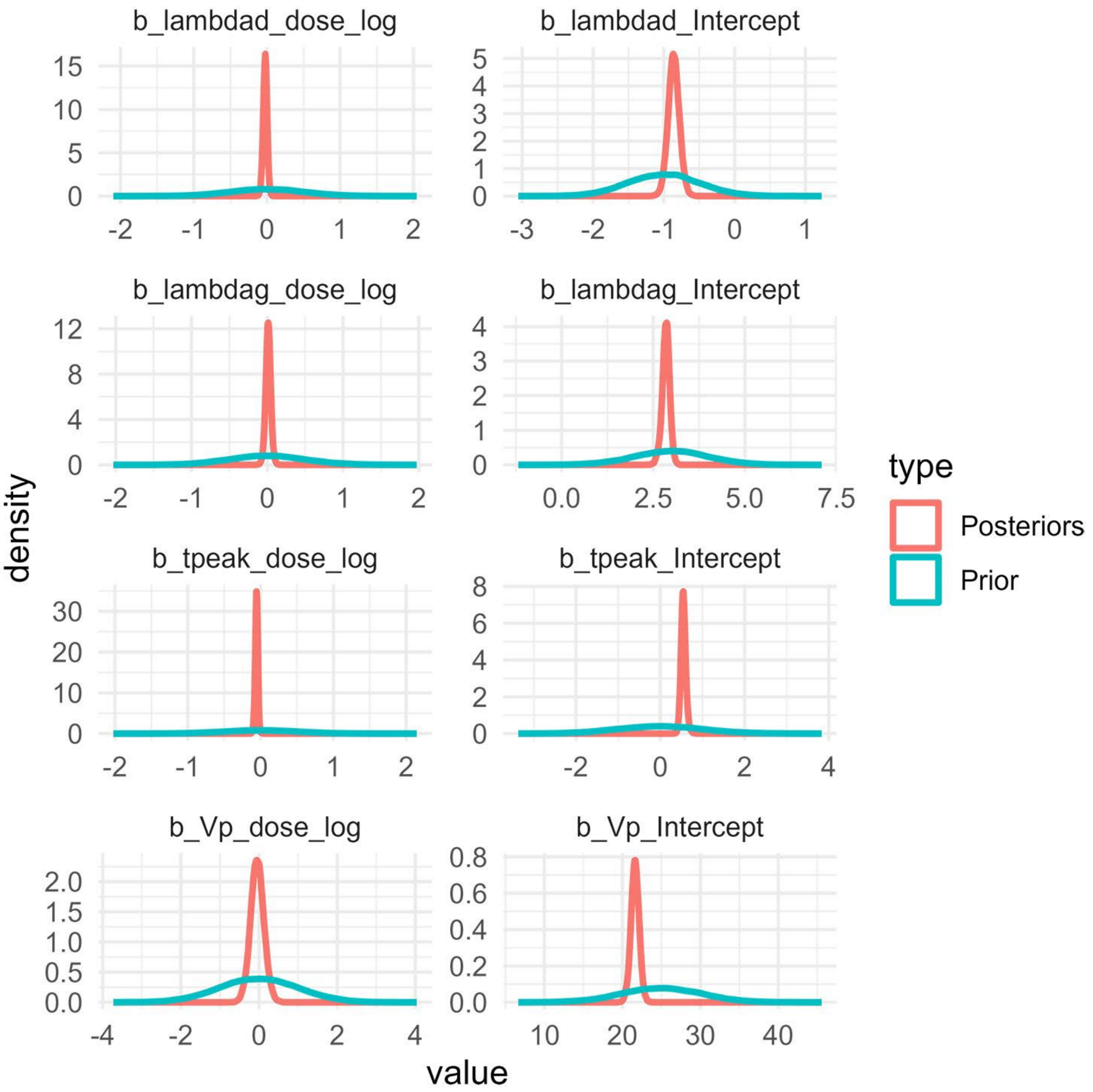

Appendix Figure 3. Comparisons of parameters' prior and posterior distributions for the model with one the very-low dose infected individual.

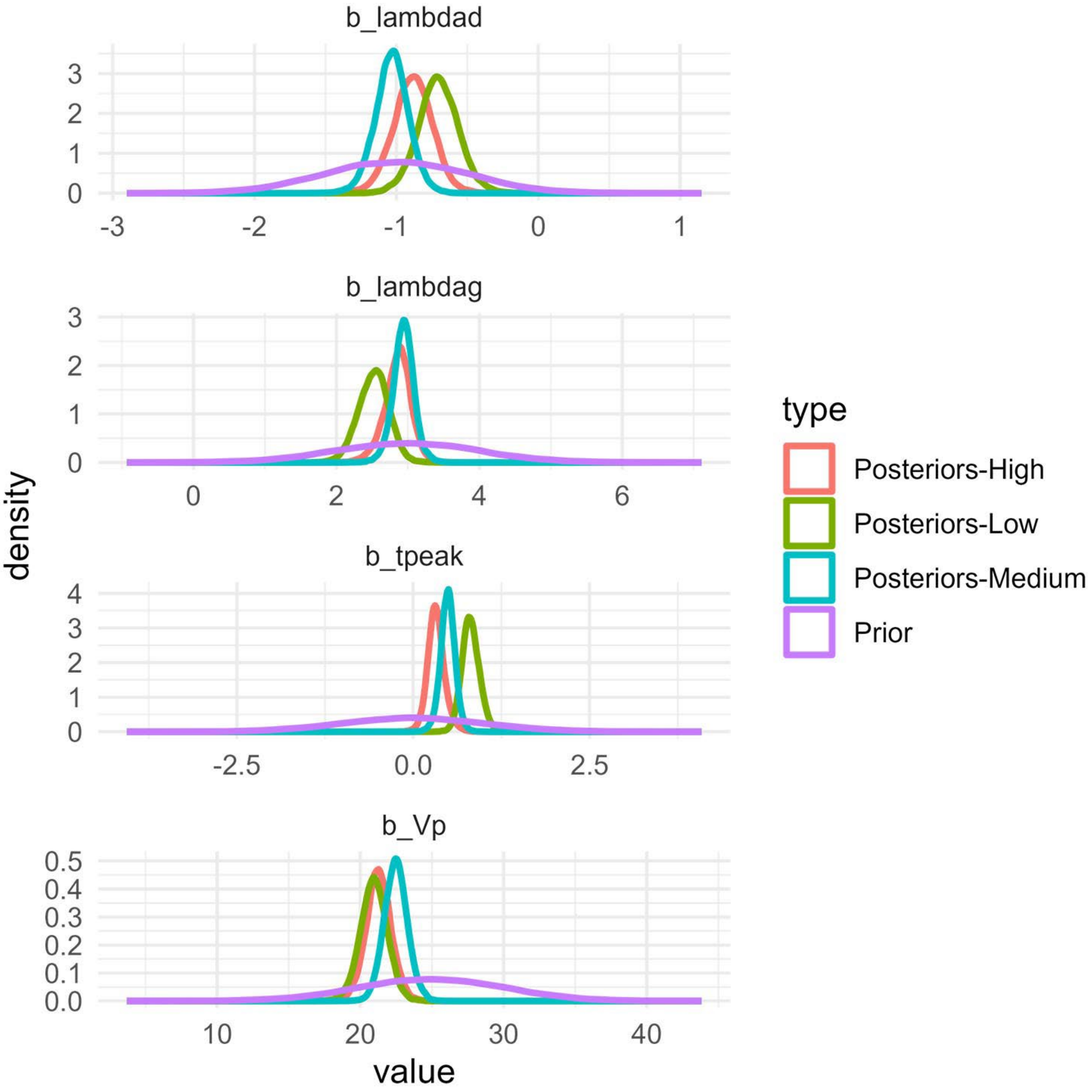

Appendix Figure 4. Comparisons of parameters' prior and posterior distributions for the model dose as a categorical variable.

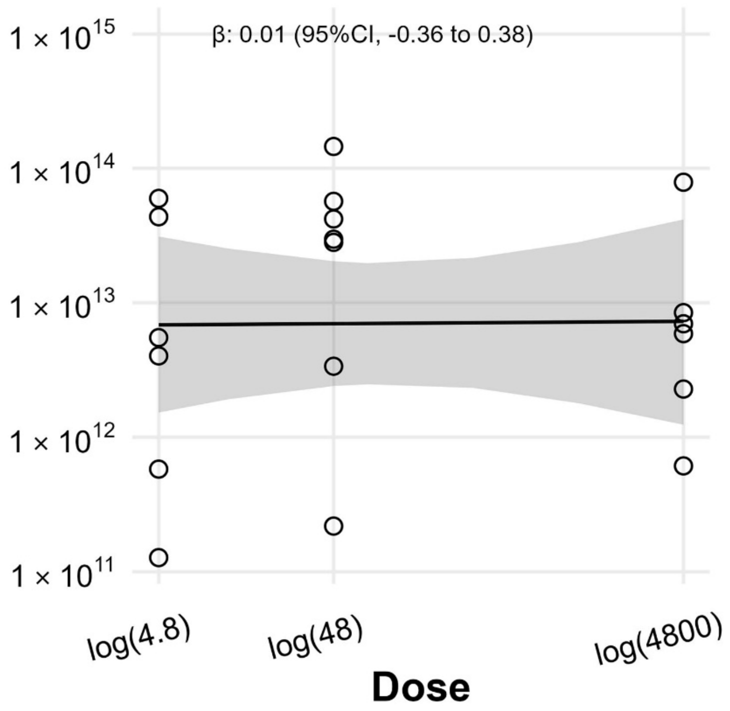

Appendix Figure 5. Virus shedding in feces during the full observation period. Everything else as described for main text Appendix Figure 1.

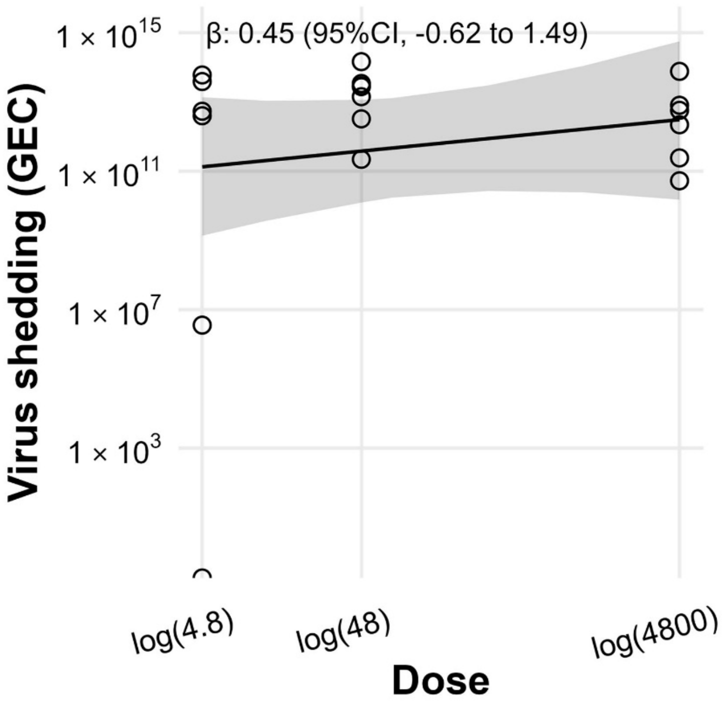

Appendix Figure 6. The association between dose and virus shedding in feces (first 4 days) when values below the LOD1 were set to 1, and values between LOD1 and LOD2 were set to 15000 (the LOD1 value).

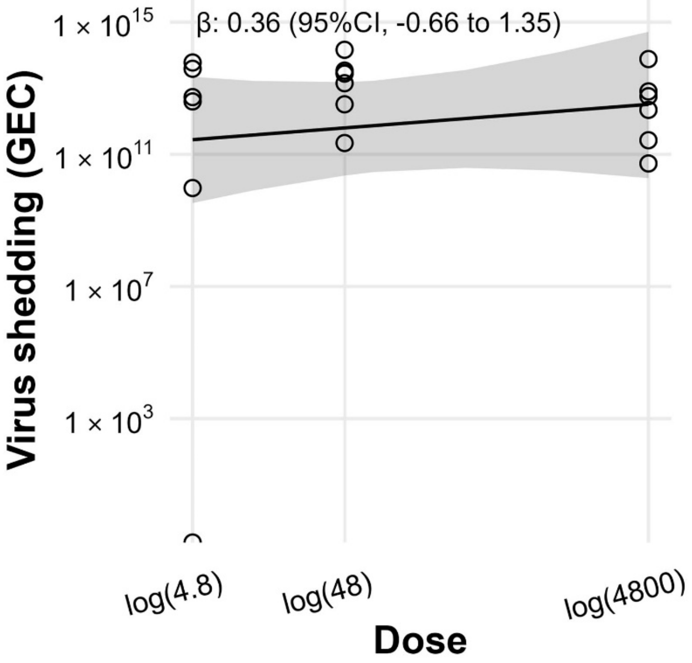

Appendix Figure 7. The association between dose and virus shedding in feces (first 4 days) when values below the LOD1 were set to 1, and values between LOD1 and LOD2 were set to 4e7 (the LOD2 value).

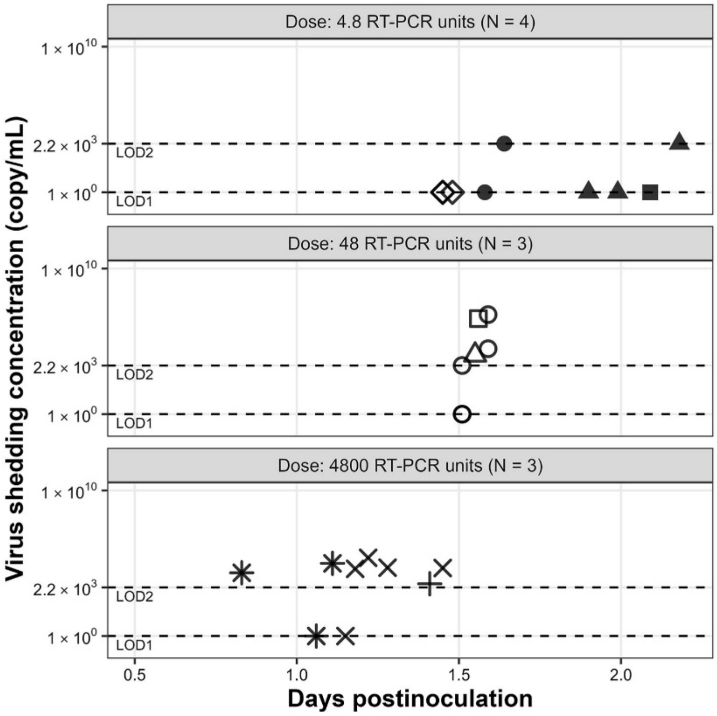

Appendix Figure 8. Vomiting events and virus concentration for each event, stratified by dose. Symbols indicate different individuals. 4, 3 and 3 individuals had at least one vomiting event in the low/medium/high dose groups respectively.

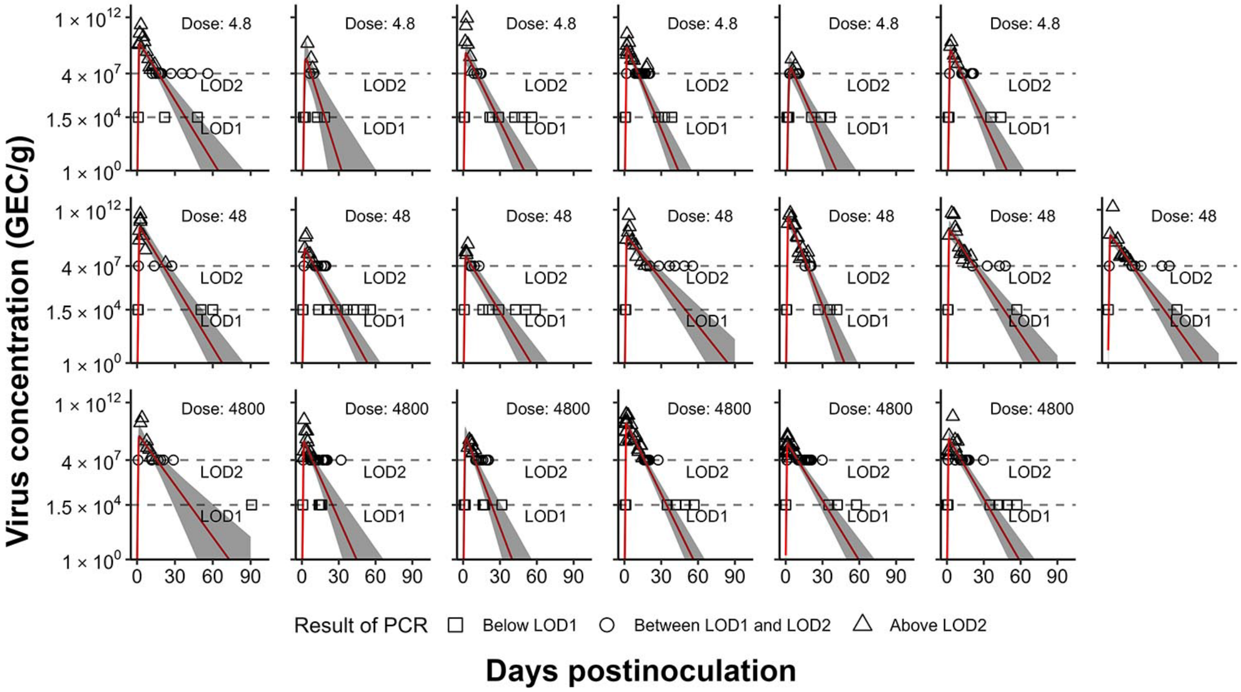

Appendix Figure 9. Fitted fecal virus concentration curves. The red lines show mean of estimations. The gray areas show 95% equal-tailed credible interval (CI). LOD1 and LOD2 indicate the two limits of detection. Points with triangle shape represented samples that are positive qRT-PCR. Points with circle shape represented samples that are positive IMC and negative qRT-PCR. Points with square shape represented samples that are negative IMC and negative qRT-PCR. Data are shown as originally reported, values below either of the LODs were treated during model fitting as described above.

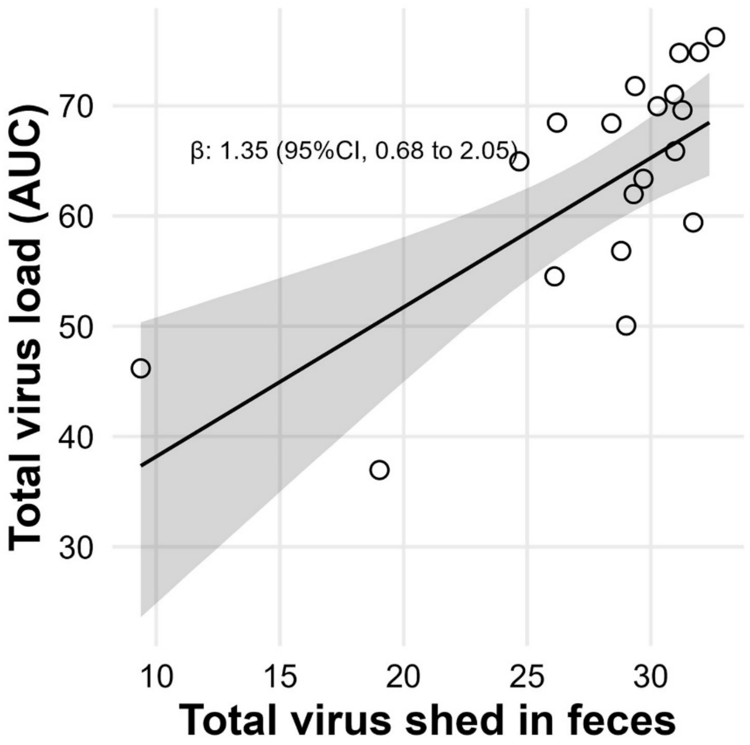

Appendix Figure 10. Correlation plot between total virus shed in feces and virus load. Open circles represent data points. The lines and shaded regions indicate the mean and 95% credible intervals of the fitted Bayesian model.

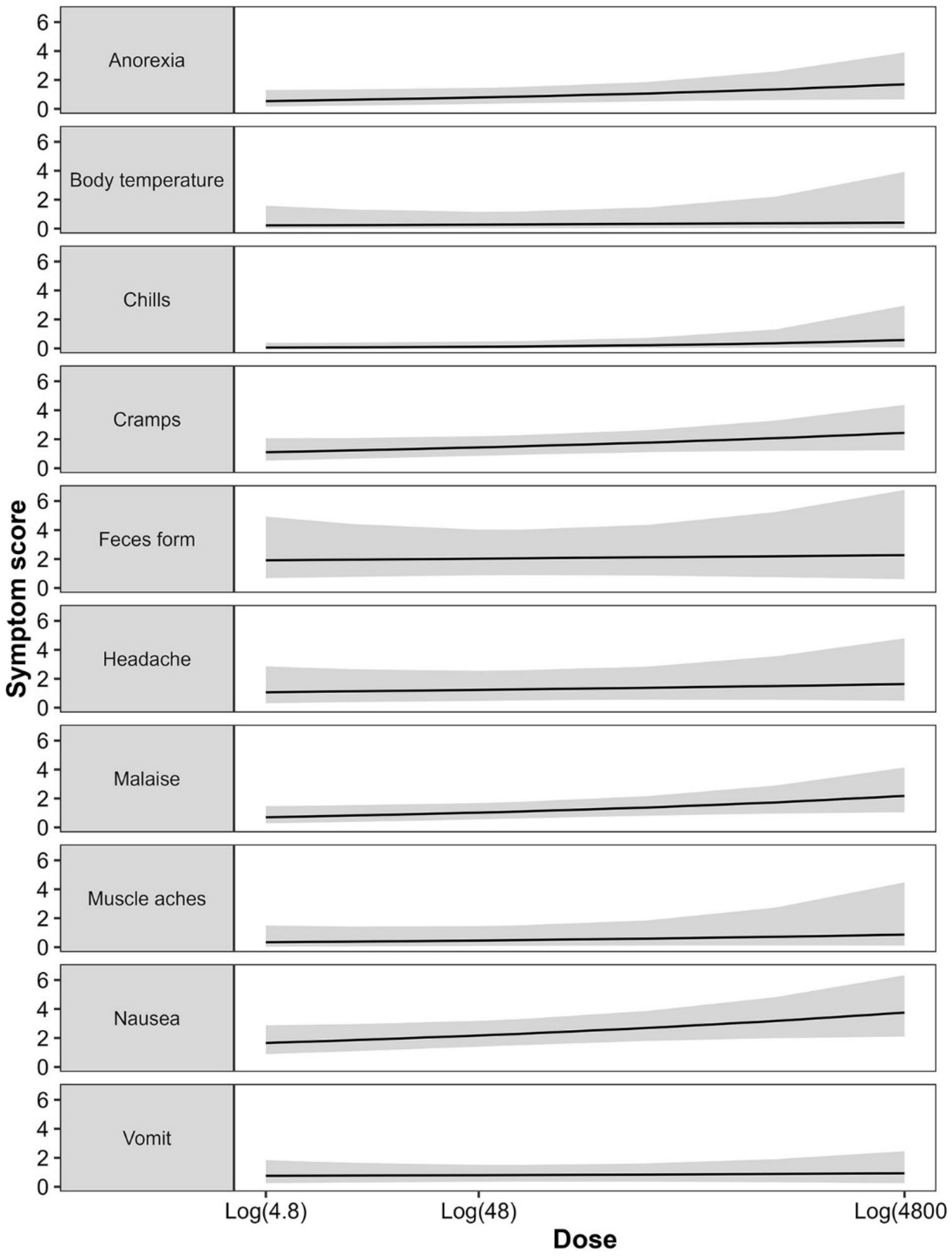

Appendix Figure 11. Associations between dose and each type of symptoms used for the calculation of comprehensive symptom score. The impact of dose was tested with each kind of symptom in the CSS.

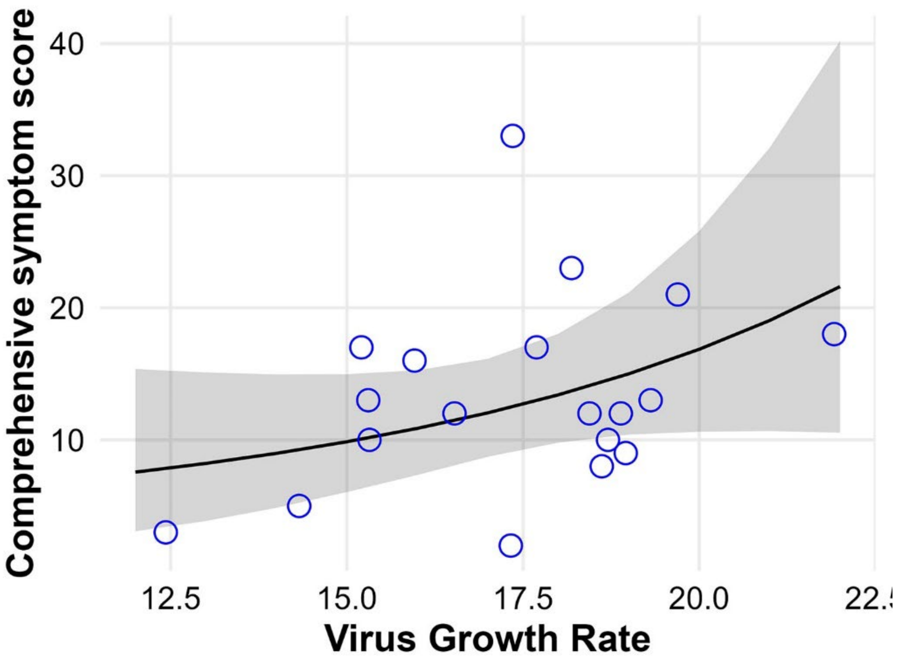

Appendix Figure 12. Associations between CSS and virus growth rate estimated by the time series model.

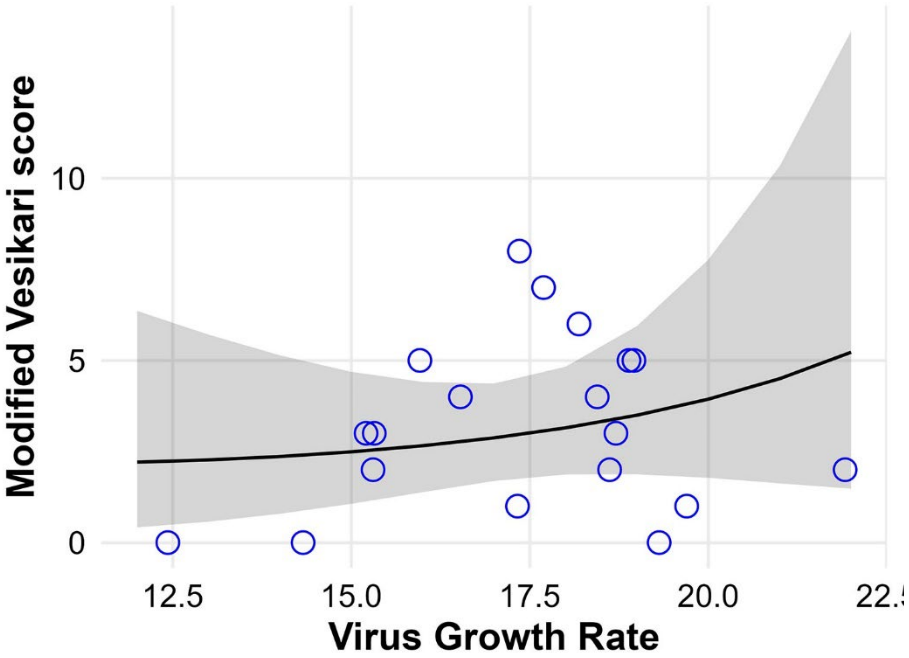

Appendix Figure 13. Associations between MVS and virus growth rate estimated by the time series model.

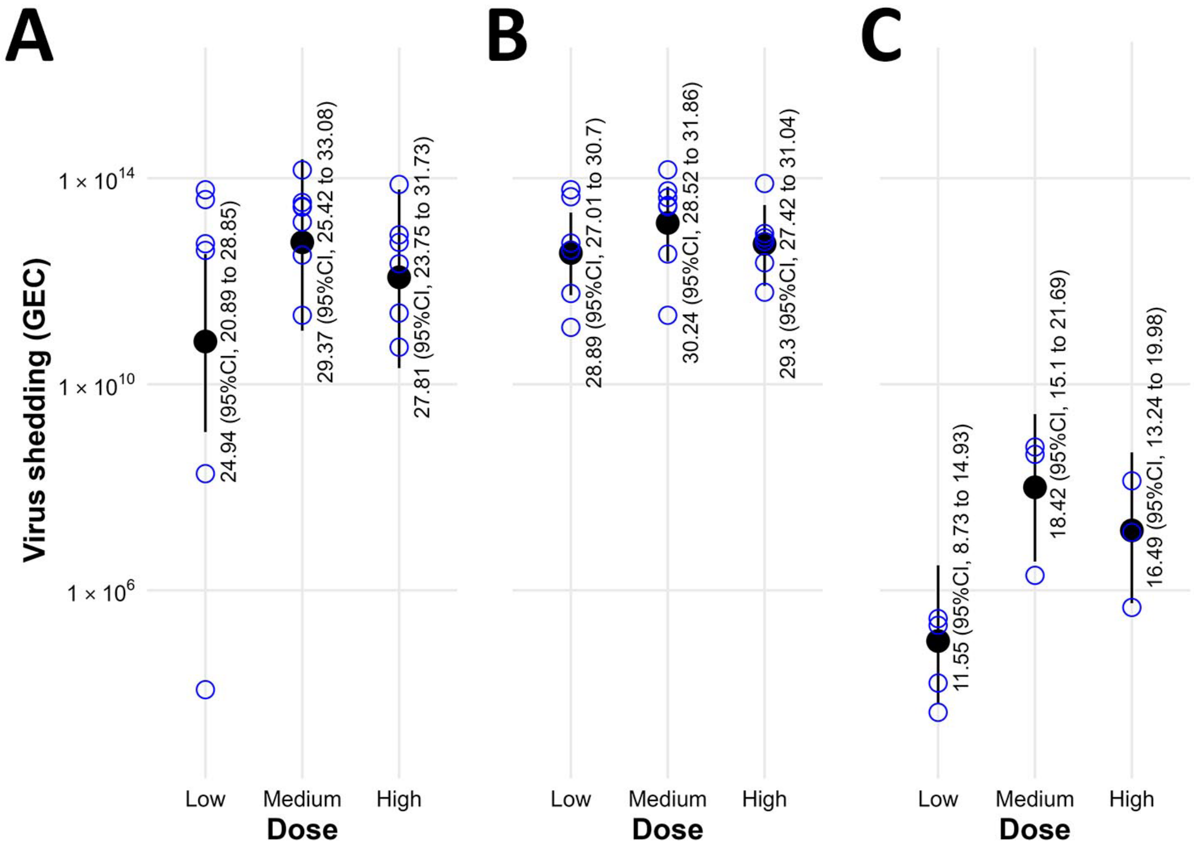

Appendix Figure 14. Virus shedding in feces or vomit. The bars show 95% equal-tailed credible intervals (CI). Points with circle shape are raw data. A) Fecal virus shedding in the first 96 hours. B) Fecal virus shedding with all data. C) Vomit virus shedding. The points show mean of estimations. Missing values due to LODs were replaced with fixed values (Supplementary, accounting for limits of detection).

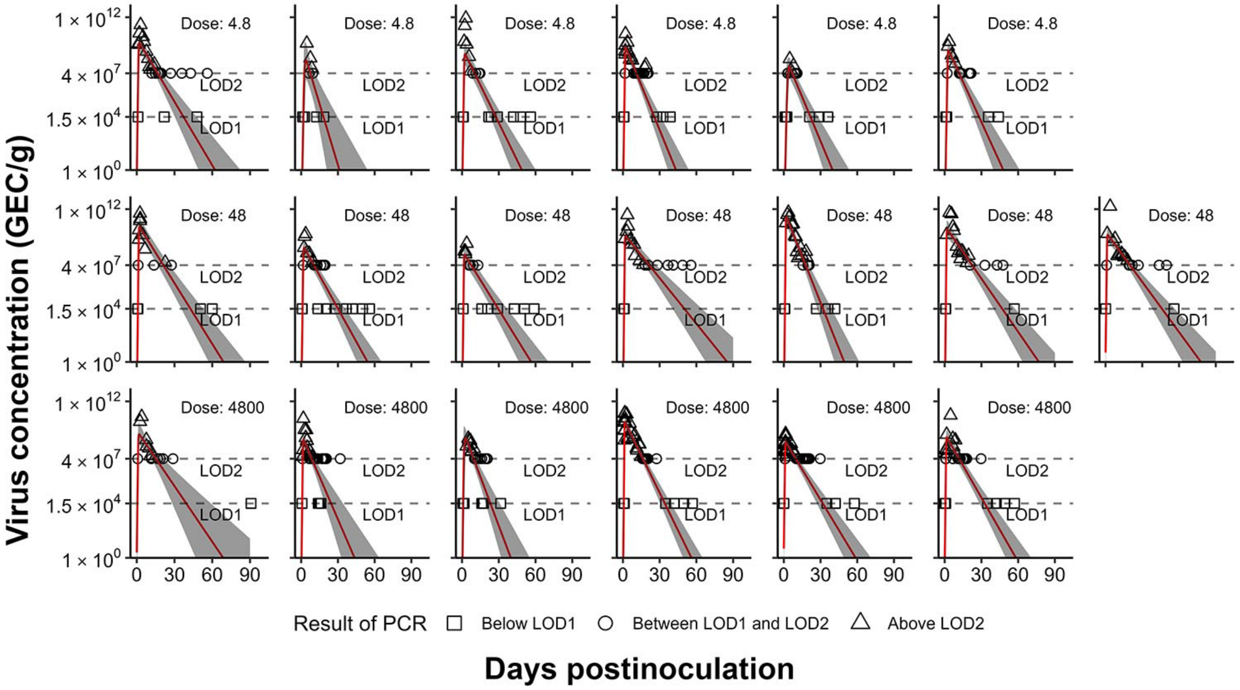

Appendix Figure 15. Fitted fecal virus concentration curves. The red lines show means of estimations. The gray areas show 95% equal-tailed credible intervals (CI). LOD1 and LOD2 indicate the two limits of detection. Points with triangle shape represented samples that with positive qRT-PCR results. Points with circle shape represented samples with positive IMC and negative qRT-PCR results. Points with square shape represented samples with negative IMC and negative qRT-PCR results. Missing values due to LODs were treated as censors (Supplementary accounting for limits of detection).

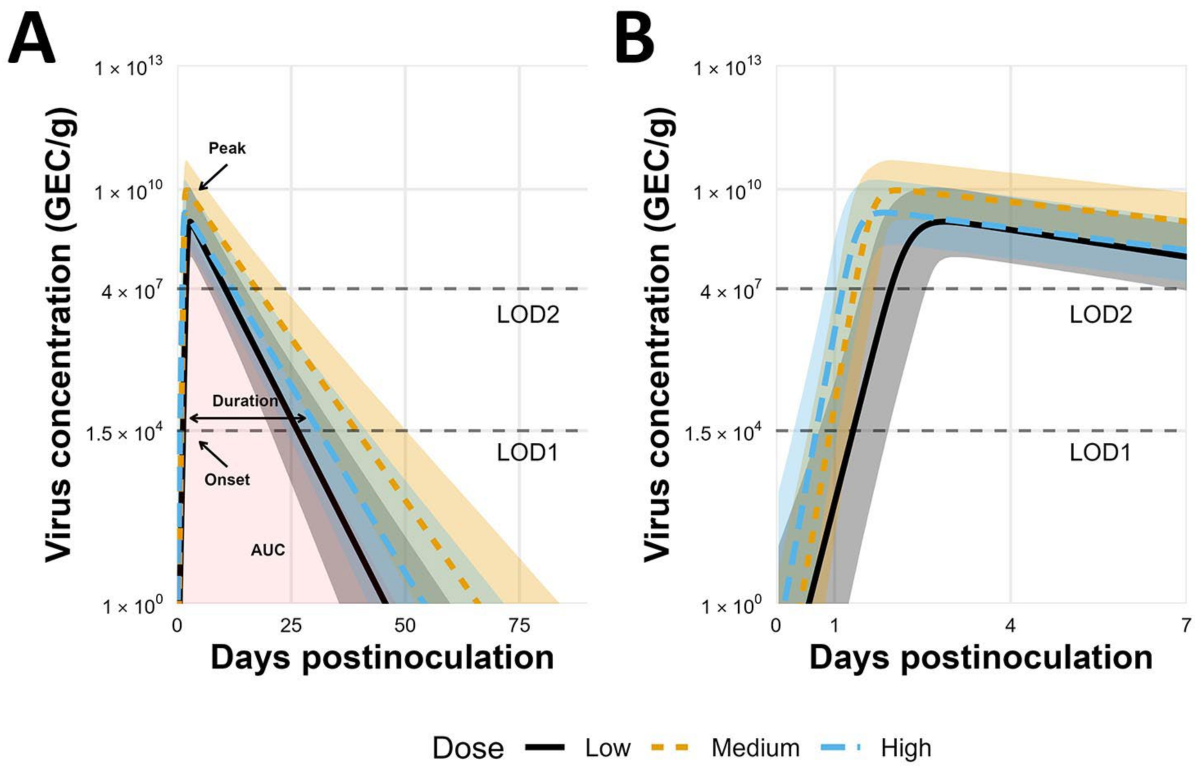

Appendix Figure 16. Fitted virus concentration (GEC/g) in feces. The lines show means of estimations. The colored areas show 95% equal-tailed credible intervals (CI). LOD1 and LOD2 represent the two limits of detection. A) The fitted curves for 90 days. B) The fitted curves for the first 7 days. Missing values due to LODs were treated as censors (Supplementary accounting for limits of detection).

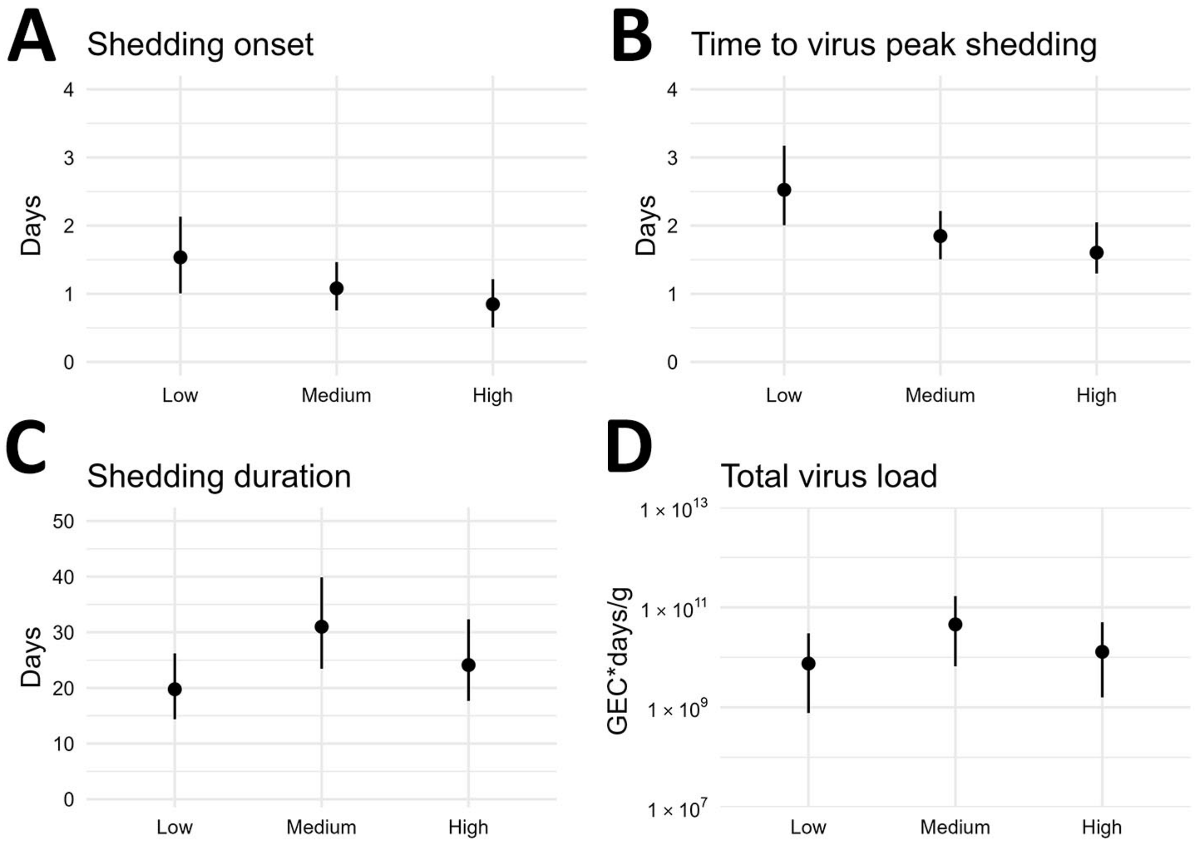

Appendix Figure 17. Model predictions for viral kinetics as a function of inoculum dose. The points show means of estimations. The bars show 95% equal-tailed credible intervals (CI). A) Time to detection (above $15{,}000$ GEC). B) Time to peak. C) Shedding duration (period that virus concentration above $15{,}000$ GEC). D) Total virus load (area under concentration curve).

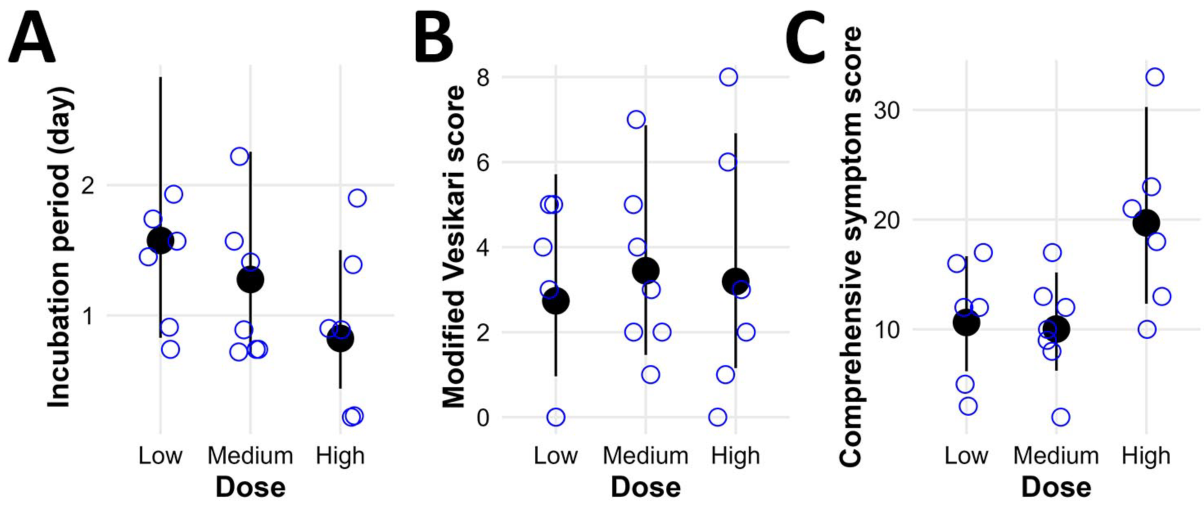

Appendix Figure 18. Estimated dose impact on symptoms. The points show means of estimations. The bars show 95% equal-tailed credible intervals (CI). Points with circle shape are raw data.

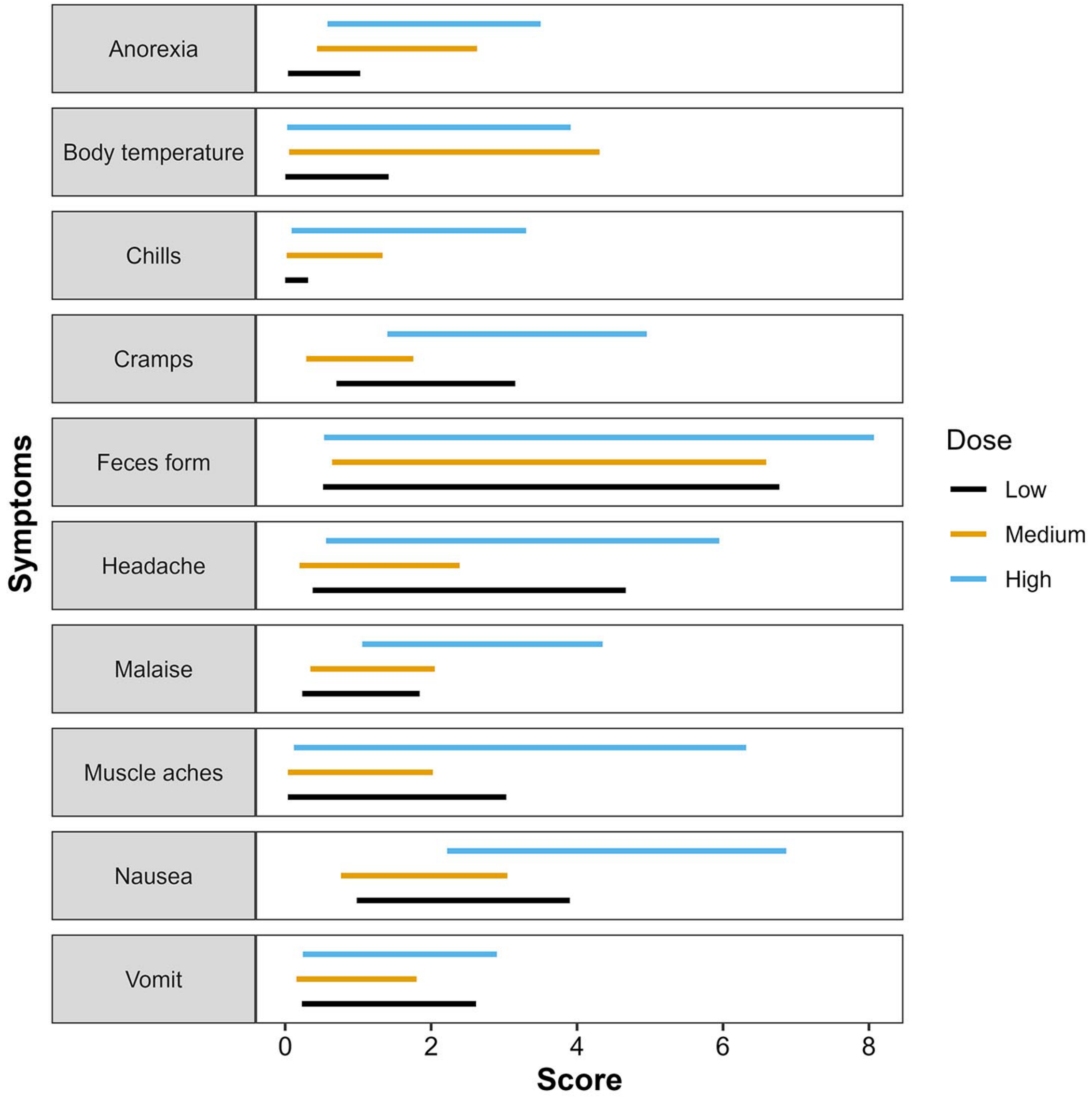

Appendix Figure 19. Associations between dose and each type of symptoms used for the calculation of comprehensive symptom score. The impact of dose was tested with each kind of symptom in the CSS.

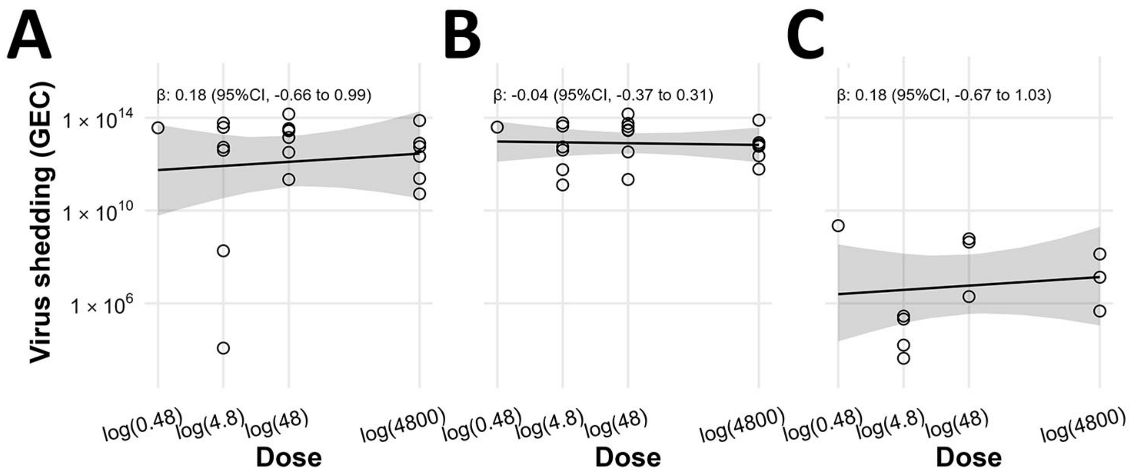

Appendix Figure 20. Virus shedding in feces or vomit. A) Total fecal virus shedding in the first 96 hours. B) Total fecal virus shedding with all data. C) Total vomit virus shedding. The lines show means of estimations. The gray areas show 95% equal-tailed credible intervals (CI). Points with circle shape are raw data. Missing values due to LODs were replaced with fixed values (Supplementary, accounting for limits of detection).

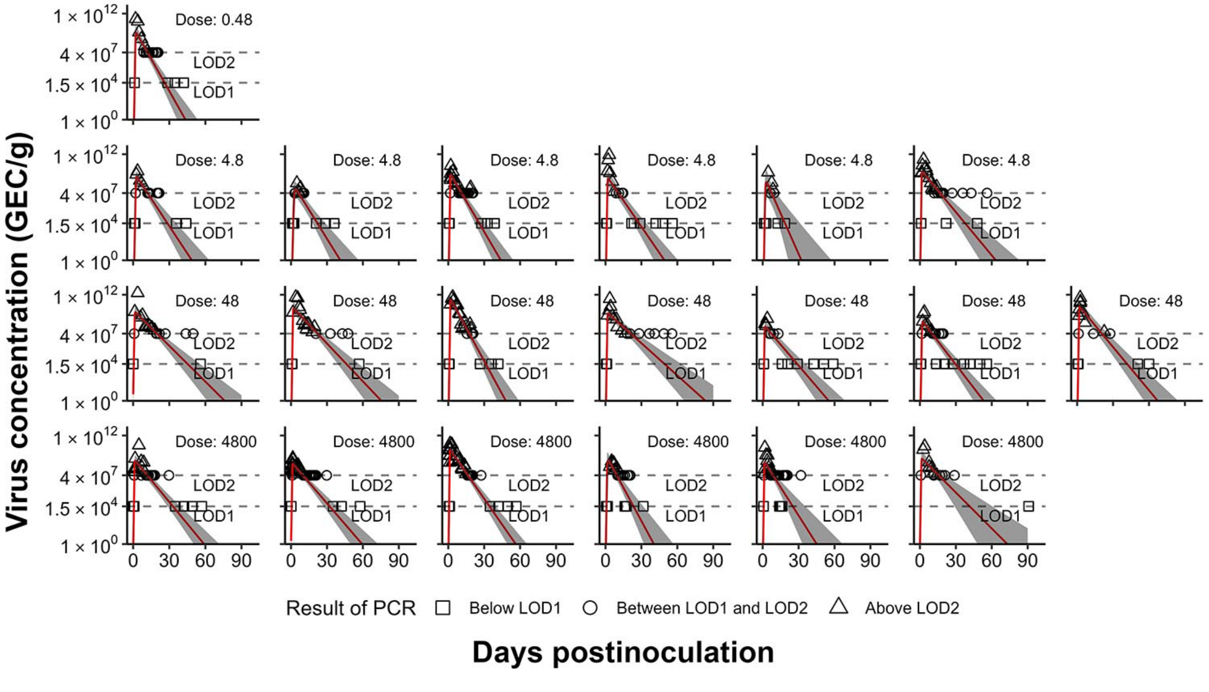

Appendix Figure 21. Fitted fecal virus concentration curves. The red lines show means of estimations. The gray areas show 95% equal-tailed credible intervals (CI). LOD1 and LOD2 indicate the two limits of detection. Points with triangle shape represent samples that are positive for qRT-PCR. Points with circle shape represented samples that are positive for IMC and negative for qRT-PCR. Points with square shape represented samples that are negative for IMC and negative for qRT-PCR. Missing values due to LODs were treated as censors (Supplementary accounting for limits of detection).

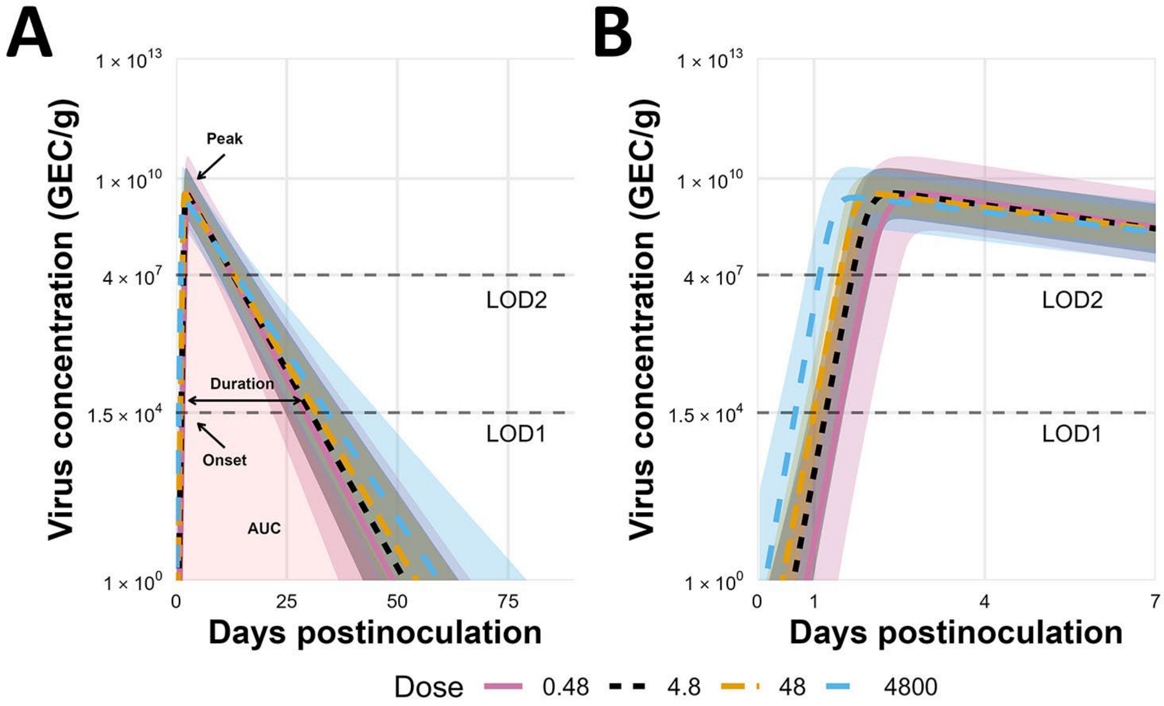

Appendix Figure 22. Fitted virus concentration (GEC/g) in feces. The lines show means of estimations. The colored areas show 95% equal-tailed credible intervals (CI). LOD1 and LOD2 represent the two limits of detection. A) The fitted curves for 90 days. B) The fitted curves for the first 7 days. Missing values due to LODs were treated as censors (Supplementary accounting for limits of detection).

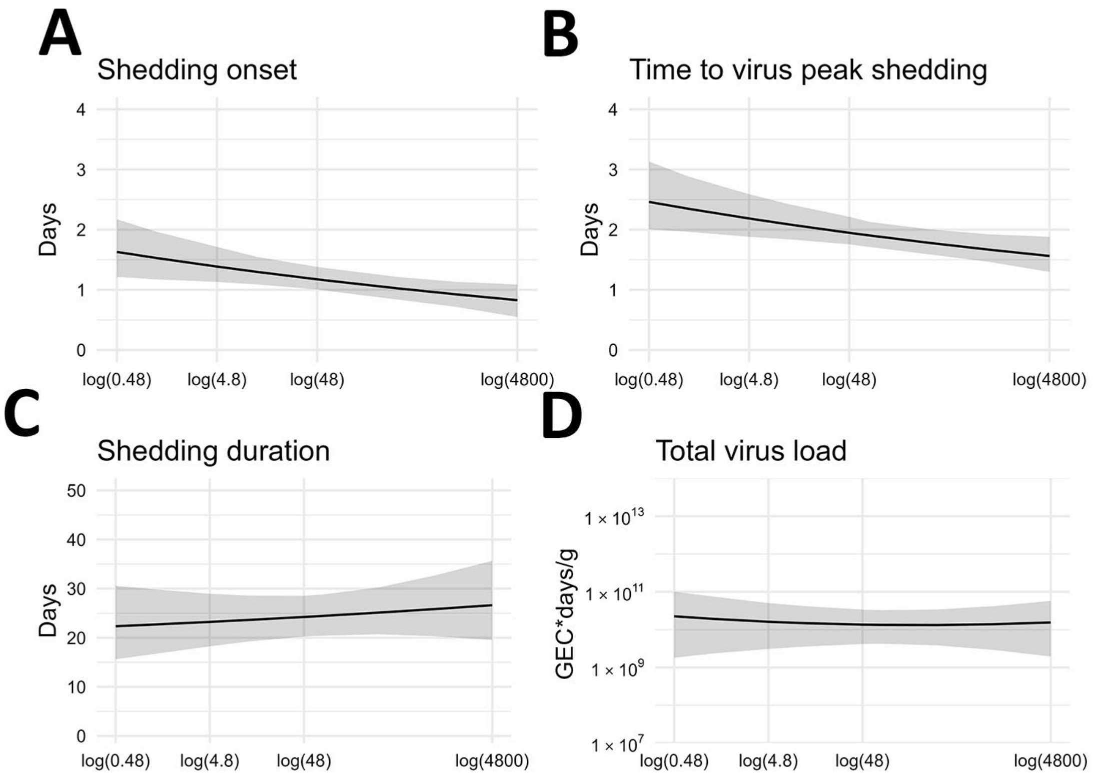

Appendix Figure 23. Model predictions for viral kinetics as a function of inoculum dose. The lines show means of estimations. The gray areas show 95% equal-tailed credible intervals (CI). A) Time to detection (above $15{,}000$ GEC). B) Time to peak. C) Shedding duration (period that virus concentration above $15{,}000$ GEC). D) Total virus load (area under curve).

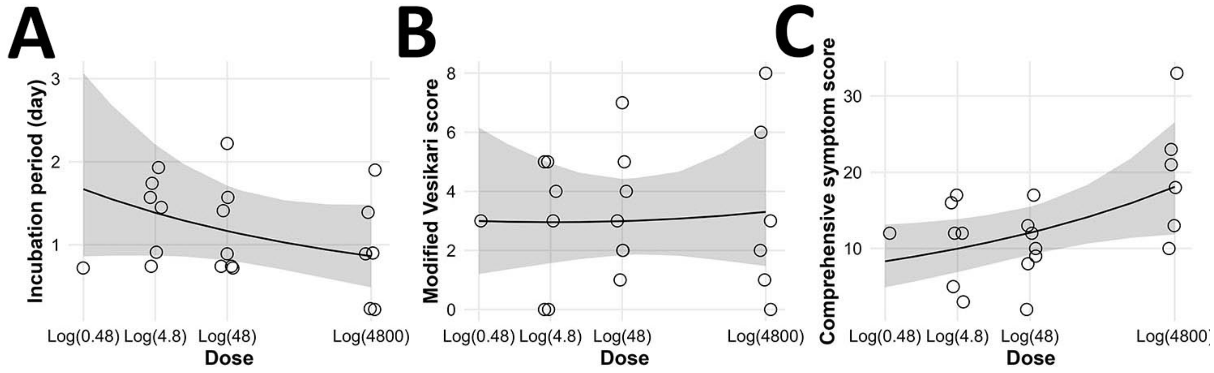

Appendix Figure 24. Estimated dose impact on symptoms. The lines show means of estimations. The gray areas show 95% equal-tailed credible intervals (CI). Points with circle shape are raw data.

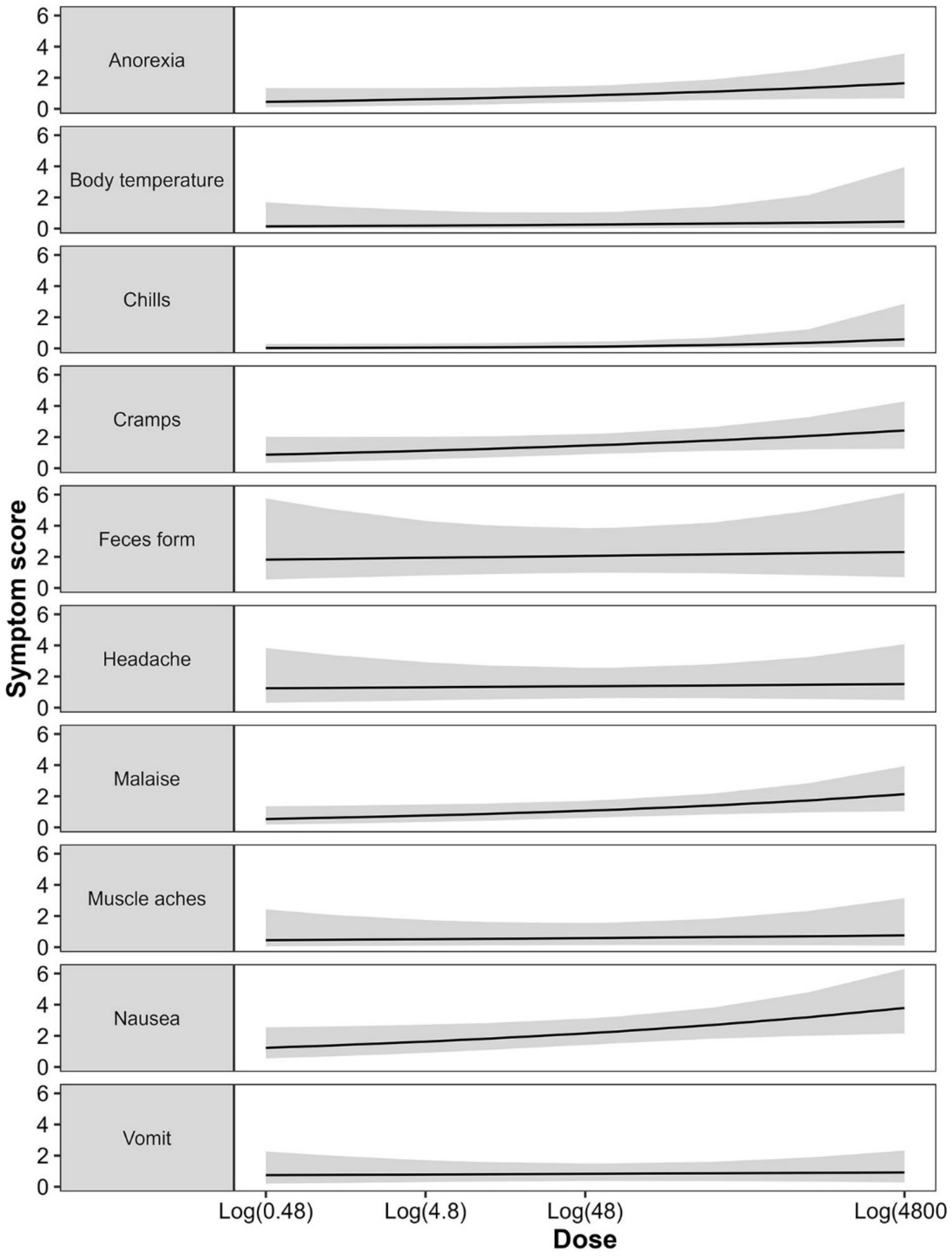

Appendix Figure 25. Associations between dose and each type of symptoms used for the calculation of comprehensive symptom score. The impact of dose was tested with each kind of symptom in the CSS.
